# Enantioselective synthesis of planar chiral ferrocenes via palladium-catalyzed annulation with diarylethynes

**DOI:** 10.3762/bjoc.9.222

**Published:** 2013-09-18

**Authors:** Yan-Chao Shi, Rong-Fei Yang, De-Wei Gao, Shu-Li You

**Affiliations:** 1 State Key Laboratory of Organometallic Chemistry, Shanghai Institute of Organic Chemistry, Chinese Academy of Sciences, 345 Lingling Lu, Shanghai 200032, China; 2 Process Development and Manufacturing Department, Pharmaron (Beijing) Co. Ltd., 6 Taihe Road, BDA, Beijing, 100176, China

**Keywords:** annulation, asymmetric catalysis, C–H activation, ferrocene, palladium, planar chirality

## Abstract

When Boc-L-Val-OH was used as a ligand for the enantioselective Pd(II)-catalyzed annulation of *N,N*-substituted aminomethyl ferrocene derivatives with diarylethynes, ferrocenes with planar chirality could be achieved with excellent enantioselectivity (up to 99% ee).

## Introduction

Chiral ferrocene derivatives have been widely applied to asymmetric catalysis, materials science, biomedical research, etc. [1–4]. Particularly, ferrocenes with planar chirality are applied as efficient ligands or catalysts in asymmetric catalysis [5–15]. However, the typical method for introduction of planar chirality in the ferrrocene backbone is still utilizing the chiral auxiliaries strategy [16–21]. Snieckus and co-workers reported the synthesis of planar chiral ferrocenes by utilizing an external chiral base such as (−)-sparteine [22–23]. Ogasawara and co-workers used the ring closing metathesis reaction to provide a novel and efficient route to synthesize the planar chiral ferrocenes [24–28]. Despite these pioneering studies, the catalytic asymmetric methods to introduce ferrocenyl planar chirality are rather limited.

Recently, a monoprotected amino acid was introduced as an efficient ligand in Pd-catalyzed enantioselective C–H activation by Yu and co-workers [29–50]. Inspired by their works, we reported a direct arylation of ferrrocene with arylboronic acid to introduce planar chirality into the ferrocene backbone using *N,N*-dimethylaminomethyl as the directing group and Boc-L-Val-OH as the ligand [51–57]. The product could be transformed into a planar chiral *P,N*-ligand, which was found to be efficient for Pd-catalyzed allylic alkylation reaction albeit with low enantioselectivity.

**Scheme 1:**
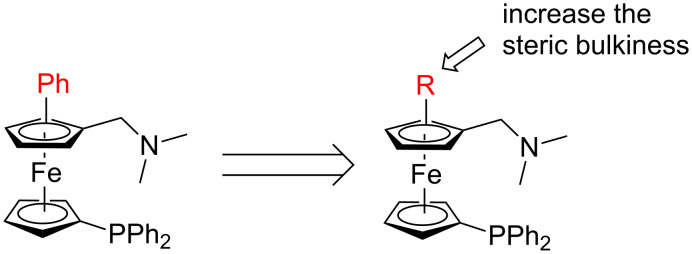
Design of the planar chiral *P*,*N*-ligand.

We envisaged that by introducing a larger substituent R in the ferrocene Cp ring would enhance the enantiocontrol of the planar chiral *P,N*-ligand (Scheme 1). Cui, Wu and their co-workers recently reported a Pd-catalyzed dehydrogenative annulation of *N,N*-dimethylaminomethylferrocene in a racemic form [58–66]. To test our hypothesis, we decided to turn such a Pd-catalyzed direct coupling of *N,N*-disubstituted aminomethylferrocenes with diarylethyne into an enantioselective reaction. Then planar chiral ferrocenyl *P,N*-ligands with a large substituent could be readily synthesized. In this paper, we report the results from this study.

## Results and Discussion

We initiated the study by testing the reaction of ferrrocene **1a** in a palladium-catalyzed direct coupling with diphenylethyne in the presence of 10 mol % Pd(OAc)_2_, 20 mol % Boc-L-Phe-OH, 25 mol % TBAB, and 100 mol % K_2_CO_3_ in DMA at 110 °C under air. To our great delight, the reaction furnished the desired product **3aa** in 28% yield and 84% ee (entry 1, Table 1). When the temperature was decreased to 80 °C, the reaction was sluggish (the ferrocene starting material was consumed in 48 h) and the enantioselectivity was improved to 93% ee (33% yield, 93% ee, entry 2, Table 1). When Fmoc-L-Phe-OH was used as the ligand, the enantioselectivity decreased dramatically (37% ee, entry 3, Table 1). Next, an array of *N*-Boc protected L-amino acids was investigated. The results are summarized in Table 1. In general, all *N*-Boc protected L-amino acids gave excellent enantioselectivity (>90% ee, entries 4–9, Table 1). Boc-L-Val-OH and Boc-L-Tle-OH were found to be the optimal chiral ligands, providing the desired product in 98% ee (entries 6 and 7, Table 1). Boc-L-Val-OH was chosen as the ligand for further studies because of the higher yield (42% yield) obtained compared with Boc-L-Tle-OH (31% yield).

**Table 1:** Examination of ligands, temperature and additives^a^.

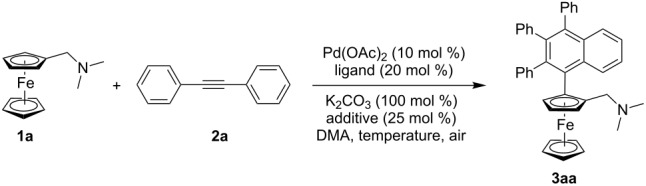						

Entry	Ligand	Additive	Temp (°C)	*t* (h)	Yield (%)^b^	ee (%)^c^

1	Boc-L-Phe-OH	TBAB	110	24	28	84
2	Boc-L-Phe-OH	TBAB	80	48	33	93
3	Fmoc-L-Phe-OH	TBAB	80	48	32	37
4	Boc-L-Ala-OH	TBAB	80	48	29	90
5	Boc-L-Abu-OH	TBAB	80	48	37	90
**6**	**Boc-L-Val-OH**	**TBAB**	**80**	**48**	**42**	**98**
7	Boc-L-Tle-OH	TBAB	80	48	31	98
8	Boc-L-Ile-OH·0.5H_2_O	TBAB	80	48	47	92
9	Boc-L-Leu-OH	TBAB	80	48	45	90
10	Ac-L-Val-OH	TBAB	80	48	33	94
11	Cbz-L-Val-OH	TBAB	80	48	18	94
12	Boc-L-Val-OH	TBAB	60	48	29	97
13	Boc-L-Val-OH	TBAB	110	24	31	85
14	Boc-L-Val-OH	TBACl	80	48	28	96
15	Boc-L-Val-OH	TBAI	80	48	39	95
16	Boc-L-Val-OH	–	80	48	29	76

^a^Reaction conditions: **1a** (0.2 mmol), **2a** (2.3 equiv), Pd(OAc)_2_ (10 mol %), ligand (20 mol %), K_2_CO_3_ (100 mol %), additive (25 mol %) in 1.5 mL DMA under air. ^b^Isolated yield. ^c^Determined by HPLC analysis. TBAB = tetrabutylammonium bromide. TBACl = tetrabutylammonium chloride. TBAI = tetrabutylammonium iodide. DMA = dimethylacetamide.

When the protecting group on the nitrogen of L-Val-OH was changed to Ac or Cbz, the enantioselectivity slightly decreased (94% ee, entries 10 and 11, Table 1). The oxidants such as Cu(OAc)_2_, Cu(OTf)_2_, Ag_2_CO_3_, Ag_2_O, AgOAc and benzoquinone (BQ) were examined but none of them could improve the yield efficiently (for details, see Supporting Information File 1). Lowering the reaction temperature to 60 °C, excellent enantioselectivity (97% ee) could be obtained, but with a decreased yield as the starting material was not fully consumed (entry 12, Table 1). When TBACl (entry 14, Table 1) or TBAI (entry 15, Table 1) was used instead of TBAB as the additive, excellent enantioselectivity was maintained. The enantioselectivity decreased dramatically when no additive was used (entry 16, Table 1). The optimized conditions were obtained as the following: 10 mol % Pd(OAc)_2_, 2.3 equiv of diarylethyne, 20 mol % Boc-L-Val-OH, 100 mol % K_2_CO_3_ and 25 mol % TBAB in DMA at 80 °C under air (entry 6, Table 1). To be noted, the yields reported in the corresponding racemic study [58] in general are higher; however, these results are not reproduced in our hands. In our studies, although the ferrocene starting material was fully consumed in most of the cases, the sensitivity of ferrocene derivatives toward oxidation conditions might lead to the low yields.

With the above mentioned optimized conditions, various aminomethylferrocene derivatives and diarylethynes were tested to evaluate the scope of this reaction. The results are given in Table 2. Various substituted diarylethynes with either an electron-donating group or an electron-withdrawing group were tolerated providing the corresponding products in 28–45% yields with 92–99% ee. All the reactions gave excellent enantioselectivity but moderate yields. When a diarylethyne bearing a 4-methoxy group was used, the yield was relatively higher (**3ac**, 45% yield; **3bc**, 41% yield; **3cc**, 40% yield). To broaden the scope of this methodology, alkyl groups on nitrogen atom were also varied (**3ba**, 35% yield, 95% ee; **3bb**, 30% yield, 97% ee; **3bc**, 41% yield, 97% ee). Interestingly, when 1-[(*N*,*N*-dimethylamino)methyl]-1’-bromoferrocene (**1c**), with a bromine atom at the second Cp ring, was used, the annulation reaction could proceed smoothly (28–42% yields, 92–96% ee).

**Table 2:** Enantioselective synthesis of planar chiral ferrocenes via C–H activation^a^.

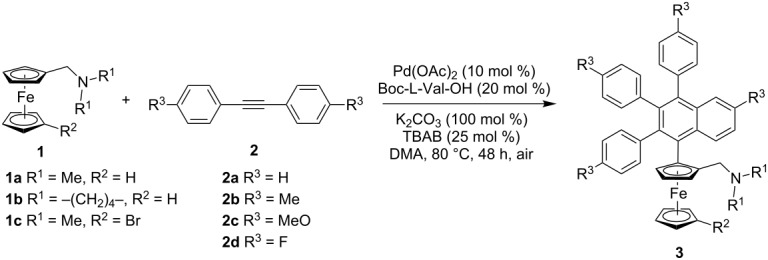					

Entry	**1**	**2**	**3**	Yield (%)^b^	ee (%)^c^

1	**1a**	**2a**	**3aa**	42	98
2	**1a**	**2b**	**3ab**	35	97
3	**1a**	**2c**	**3ac**	45	99
4	**1a**	**2d**	**3ad**	31	97
5	**1b**	**2a**	**3ba**	35	95
6	**1b**	**2b**	**3bb**	30	97
7	**1b**	**2c**	**3bc**	41	97
8	**1c**	**2a**	**3ca**	42	96
9	**1c**	**2b**	**3cb**	28	96
10	**1c**	**2c**	**3cc**	40	96
11	**1c**	**2d**	**3cd**	30	92

^a^Reaction conditions: **1** (0.2 mmol or 0.3 mmol), **2** (2.3 equiv), Pd(OAc)_2_ (10 mol %), Boc-L-Val-OH (20 mol %), K_2_CO_3_ (100 mol %), TBAB (25 mol %) in 1.5 mL DMA at 80 °C under air. ^b^Isolated yield. ^c^Determined by HPLC analysis.

**Scheme 2:**
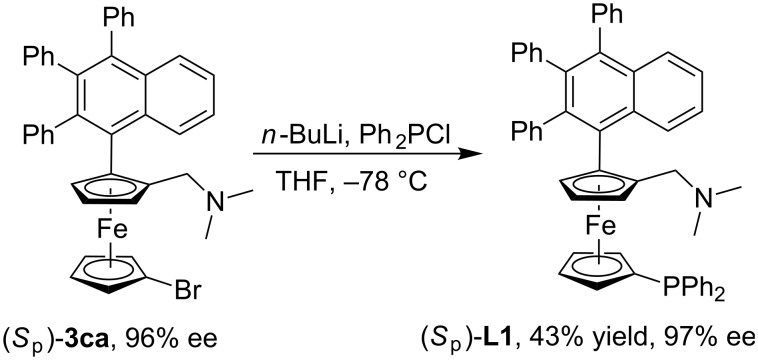
Synthesis of planar chiral *P*,*N*-ligand (*S*_p_)-**L1**.

**Scheme 3:**
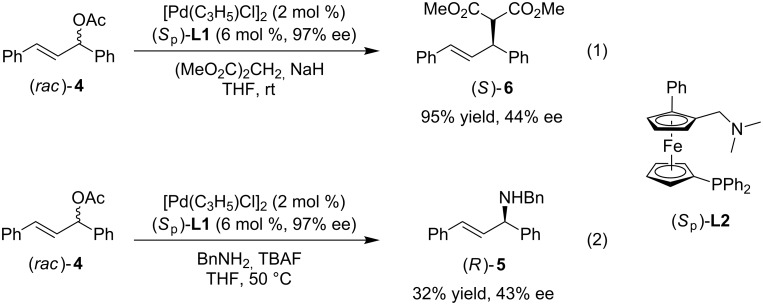
Pd-catalyzed asymmetric allylic alkylation and amination reactions with (*S*_p_)-**L1**.

The absolute configuration of the products was assigned as *S*_p_ from the cyclopalladated complex described in the literature and our previous study [51,67–68]. Next, to test our original hypothesis, planar chiral *P*,*N*-ligand (*S*_p_)-**L1** was prepared from (*S*_p_)-**3ca**. Starting from (*S*_p_)-**3ca** (96% ee), lithiation with *n*-BuLi followed by quenching with Ph_2_PCl gave the planar chiral *P*,*N*-ligand (*S*_p_)-**L1** in 43% yield and 97% ee (Scheme 2). The allylic substitution reactions of (*rac*)-**4** had been carried out. The allylic alkylation reaction proceeded in 95% yield and 44% ee (Scheme 3, reaction 1) and the allylic amination reaction proceeded in 32% yield and 43% ee (Scheme 3, reaction 2) [69–71]. Although only moderate enantioselectivity was obtained, significant increase of enantioselectivity was obtained comparing with 15% ee obtained by (*S*_p_)-**L2** in allylic alkylation reaction [51]. The results indicated that the planar *P*,*N*-ligand with a larger R group could improve the enantioselectivity.

## Conclusion

In summary, we reported a highly enantioselective synthesis of planar chiral ferrocenes via palladium-catalyzed direct annulation of *N*,*N*-disubstituted aminomethylferrocene derivatives with diarylethynes. The commercially available *N*-Boc-L-Val-OH is an efficient ligand with air as a suitable oxidant. The planar chiral ferrocenes could be transformed readily into a *P*,*N*-ligand, which was found to be suitable for Pd-catalyzed allylic substitution reactions.

## Experimental

### General procedure for the enantioselective synthesis of planar chiral ferrocenes

To a solution of alkyne **2** (0.46 mmol) in DMA (1.5 mL) was added Boc-L-Val-OH (8.7 mg, 0.04 mmol), Pd(OAc)_2_ (4.5 mg, 0.02 mmol), K_2_CO_3_ (27.6 mg, 0.2 mmol), TBAB (tetrabutyl ammonium bromide) (16.1 mg, 0.05 mmol) and ferrocene **1** (0.02 mmol) successively. The mixture was stirred at 80 °C under air (open flask) for 48 h. After the reaction was complete, it was quenched with saturated aqueous NaHCO_3_ solution and extracted with EtOAc for three times. The combined organic layers were washed with H_2_O and brine successively, then dried over anhydrous Na_2_SO_4_ and filtrated. After the solvent was removed under reduced pressure, the residue was purified by silica gel column chromatography (ethyl acetate/petroleum ether 1:10, v/v, 3% Et_3_N) to afford the desired product **3**.

## Supporting Information

File 1 Experimental, characterization data and spectra.
