# Supplementary material for: Enantioselective synthesis of planar chiral ferrocenes via palladium-catalyzed annulation with diarylethynes
Source: Beilstein J Org Chem. 2013 Sep 18;9:1891–6. doi: 10.3762/bjoc.9.222 (PMC3778326; doi:10.3762/bjoc.9.222)
Supplement: File 1 — Experimental, characterization data and spectra. [file Beilstein_J_Org_Chem-09-1891-s001.pdf]

Supporting Information

for

Enantioselective synthesis of planar chiral ferrocenes via palladium-catalyzed annulation with diarylethynes

Yan-Chao Shi[1], Rong-Fei Yang[1,2], De-Wei Gao[1] and Shu-Li You*[1]

Address: [1]State Key Laboratory of Organometallic Chemistry, Shanghai Institute of Organic Chemistry, Chinese Academy of Sciences, 345 Lingling Lu, Shanghai 200032, China and [2]Process Development and Manufacturing Department, Pharmaron (Beijing) Co. Ltd., 6 Taihe Road, BDA, Beijing, 100176, China

Email: Shu-Li You* - E-mail: slyou@sioc.ac.cn

* Corresponding author

Experimental, characterization data and spectra.

Content

General methods	S3
Complete optimization data	S4
Enantioselective synthesis of planar chiral ferrocenes	S4
Synthesis of (S_p)-**L1**	S15
Pd-catalyzed allylic amination reaction	S16
Pd-catalyzed allylic alkylation reaction	S17
References	S17
Copies of NMR spectra and HPLC chromatographs	S19

General Methods. Unless stated otherwise, all reactions were carried out in flame-dried glassware under a dry argon atmosphere. All solvents were purified and dried according to standard methods prior to use. ^{1}H and ^{13}C NMR spectra were recorded on a Varian instrument (300 MHz and 75 MHz, 400 MHz and 100 MHz, respectively) and internally referenced to tetramethylsilane signal or residual protio solvent signals. Data for ^{1}H NMR are recorded as follows: chemical shift (δ, ppm), multiplicity (s = singlet, d = doublet, t = triplet, m = multiplet or unresolved, br = broad singlet, coupling constant(s) in Hz, integration). Data for ^{13}C NMR are reported in terms of chemical shift (δ, ppm).

Compounds **1a–c** [1-3] were prepared by reductive amination of ferrocene aldehyde with the corresponding amines. Compound **2a** was purchased from Alfa. Compounds **2b–d** were synthesized following the reported procedures [4].

Complete optimization data

Table S1: Examination of oxidants[a]

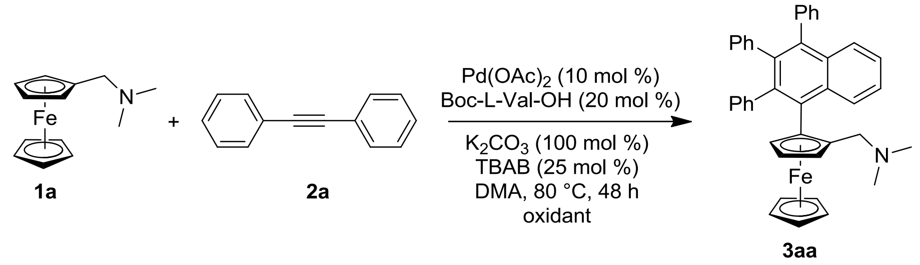

Entry	Oxidant	Yield (%)[b]	ee (%)[c]
1	air	42	98%
2	$Cu(OAc)_2$	trace	-
3	$Cu(OTf)_2$	trace	-
4	Ag_2CO_3	<5	-
5	Ag_2O	<5	-
6	AgOAc	<5	-
7	BQ	14	-

[a]Reaction conditions: **1a** (0.2 mmol), **2a** (2.3 equiv), $Pd(OAc)_2$ (10 mol %), Boc-L−Val-OH (20 mol %), K_2CO_3 (100 mol %), TBAB (25 mol %) and oxidant (2 equiv for entries 2–7 under argon) in 1.5 mL DMA. [b]Isolated yield. [c]Determined by HPLC analysis.

General procedure for the enantioselective synthesis of planar chiral ferrocenes

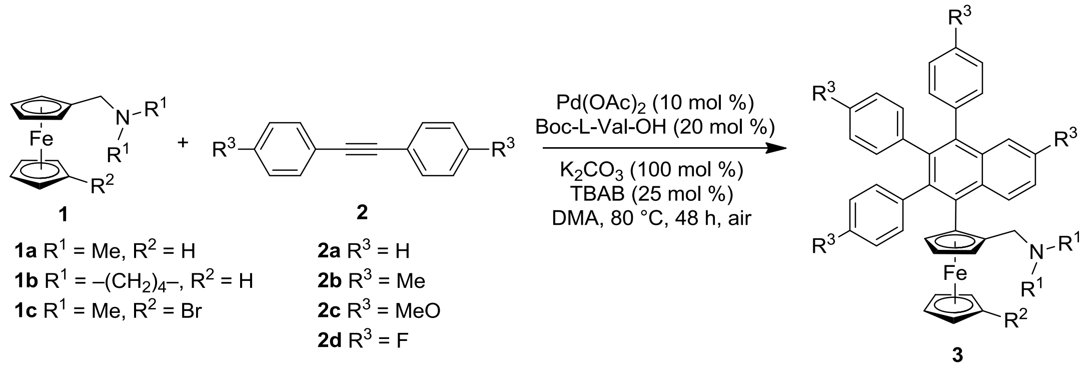

To a solution of alkyne **2** (0.46 mmol) in DMA (1.5 mL) was added Boc-L-Val-OH (8.7 mg, 0.04 mmol), $Pd(OAc)_2$ (4.5 mg, 0.02 mmol), K_2CO_3 (27.6

mg, 0.2 mmol), TBAB (tetrabutyl ammonium bromide) (16.1 mg, 0.05 mmol) and ferrocene **1** (0.02 mmol) successively. The mixture was stirred at 80 °C under air (open flask) for 48 h. After the reaction was complete, it was then quenched with saturated aqueous $NaHCO_3$ solution and extracted with EtOAc for three times. The combined organic layers were washed with H_2O and brine successively, then dried over anhydrous Na_2SO_4 and filtrated. After the solvent was removed under reduced pressure, the residue was purified by silica gel column chromatography (ethyl acetate/petroleum ether = 1/10, v/v, 3% Et_3N) to afford the desired product **3**.

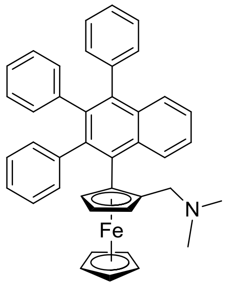

(S_P)-1-[(*N*,*N*-Dimethylamino)methyl]-2-(2,3,4-triphenylnaphthalen-1-yl)ferrocene (**3aa**)

Yellow solid (50 mg, 42% yield, 98% ee). Analytical data for **3aa**: Mp = 78–80 °C; $[\alpha]_D^{20}$ = −91.7° (*c* = 0.26 chloroform, 98% ee). ^{1}H NMR (400 MHz, $CDCl_3$) δ 2.23 (s, 6H), 3.13 (d, *J* = 14.0 Hz, 1H), 3.64 (d, *J* = 14.0 Hz, 1H), 3.73 (dd, J2 = 2.4 Hz, J_2 = 1.6 Hz, 1H), 3.85 (t, *J* = 2.1 Hz, 1H), 4.21 (s, 5H), 4.50 (dd, J_1 = 2.8 Hz, J_2 = 1.6 Hz, 1H), 6.50–6.52 (m, 1H), 6.59–6.62 (m, 1H), 6.73–6.97 (series of m, 9H), 7.09 (td, J_1 = 7.2 Hz, J_2 = 0.8 Hz, 1H), 7.18 (tt, J_1 = 7.6 Hz, J_2 = 1.2 Hz, 1H), 7.36 (td, J_1 = 7.2 Hz, J_2 = 0.8 Hz, 1H), 7.43–7.50 (m, 2H), 7.63–7.70 (m, 2H), 10.18 (d, *J* = 8.0 Hz, 1H); ^{13}C NMR (100 MHz, $CDCl_3$) δ 45.7, 58.2, 65.2, 69.1, 70.1, 71.9, 86.0, 86.9, 123.8, 125.14, 125.18, 125.7, 126.20, 126.31, 126.4, 126.6, 127.0, 127.1, 127.8, 129.0, 130.97, 131.19, 131.28, 131.39, 131.9, 132.0, 132.2, 138.4, 138.9, 139.7, 140.8, 142.1, 142.4; The enantiomeric excess was determined by phenomenex cellulose-3 (25 cm), MeOH/IPA = 90/10, 0.7 mL/min, λ = 214 nm, *t* (major) = 5.577 min, *t* (minor) = 9.827 min.

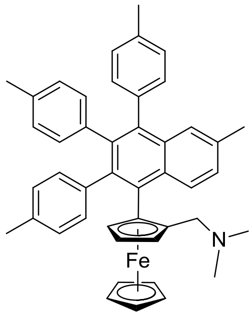

(S_P)-1-[(N,N-Dimethylamino)methyl]-2-(6-methyl-2,3,4-tri-p-tolylnaphthalen-1-yl)ferrocene (**3ab**)

Yellow solid (46 mg, 35% yield, 97% ee). Analytical data for **3ab**: Mp = 81–83 °C; $[\alpha]_D^{20}$ = −176.3° (*c* = 0.29 chloroform, 97% ee). ^{1}H NMR (300 MHz, $CDCl_3$) δ 2.10 (s, 3H), 2.14 (s, 3H), 2.22 (s, 6H), 2.33 (s, 3H), 2.45 (s, 3H), 3.05 (d, *J* = 13.8 Hz, 1H), 3.65–3.70 (m, 2H), 3.84 (t, *J* = 2.4 Hz, 1H), 4.17 (s, 5H), 4.50 (s, 1H), 6.35 (dd, J_1 = 7.8 Hz, J_2 = 1.2 Hz, 1H), 6.41 (d, *J* = 7.8 Hz, 1H), 6.55 (d, *J* = 7.8 Hz, 1H), 6.60–6.68 (m, 3H), 6.71–6.74 (m, 2H), 6.80 (dd, J_1 = 7.5 Hz, J_2 = 1.2 Hz, 1H), 6.89 (d, *J* = 8.1 Hz, 1H), 7.15 (d, *J* = 7.8 Hz, 1H), 7.33 (dd, J_1 = 7.8 Hz, J_2 = 1.5 Hz, 1H), 7.37 (s, 1H), 7.48 (dd, J_1 = 8.7 Hz, J_2 = 1.5 Hz, 1H), 10.00 (d, *J* = 9.0 Hz, 1H); ^{13}C NMR (100 MHz, $CDCl_3$) δ 21.05, 21.09, 21.3, 21.8, 45.8, 58.3, 65.1, 68.7, 69.9, 71.8, 86.3, 87.1, 125.78, 125.89, 126.98, 127.00, 127.07, 127.11, 127.7, 128.4, 129.0, 129.5, 130.8, 131.0, 131.14, 131.20, 131.31, 131.59, 131.74, 132.5, 134.0, 134.2, 134.9, 135.4, 137.0, 137.8, 138.1, 139.1, 139.3, 141.7; The enantiomeric excess was determined by phenomenex cellulose-4 (25 cm), CH_3CN/IPA = 95/5, 0.5 mL/min, λ = 254 nm, *t* (major) = 10.318 min, *t* (minor) = 9.552 min.

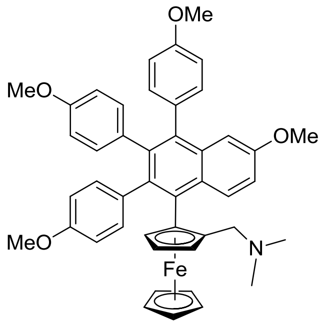

(S_P)-1-[(N,N-Dimethylamino)methyl]-2-(6-methoxy-2,3,4-tris(4-methoxyphenyl)naphthalen-1-yl)ferrocene (**3ac**)

Yellow solid (64 mg, 45% yield, 99% ee). Analytical data for **3ac**: Mp = 84–86 °C; $[\alpha]_D^{20}$ = −154.4° (*c* = 0.29 chloroform, 99% ee). ^{1}H NMR (300 MHz, $CDCl_3$) δ 2.23 (s, 6H), 3.08 (d, *J* = 13.5 Hz, 1H), 3.62 (s, 3H), 3.66 (s, 3H), 3.62–3.70 (m, 2H), 3.74 (s, 3H), 3.79 (s, 3H), 3.88 (t, *J* = 2.4 Hz, 1H), 4.17 (s, 5H), 4.51 (s, 1H), 6.30–6.47 (series of m, 6H), 6.62–6.65 (m, 2H), 6.75 (dd, J_1 = 8.4 Hz, J_2 = 2.1 Hz, 1H), 6.80 (dd, J_1 = 8.4 Hz, J_2 = 2.1 Hz, 1H), 6.90 (dd, J_1 = 8.1 Hz, J_2 = 2.4 Hz, 1H), 6.95 (d, *J* = 2.7 Hz, 1H), 7.30 (dd, J_1 = 9.3 Hz, J_2 = 2.7 Hz, 1H), 7.37 (dd, J_1 = 8.7 Hz, J_2 = 2.1 Hz, 1H), 10.06 (d, *J* = 9.3 Hz, 1H); ^{13}C NMR (100 MHz, $CDCl_3$) δ 45.5, 54.87, 54.98, 55.06, 55.11, 58.1, 65.4, 68.9, 70.0, 71.9, 86.3, 105.7, 111.86, 111.89, 112.01, 112.06, 112.7, 113.3, 115.6, 126.7, 130.7, 131.82, 132.00, 132.13, 132.17, 132.22, 132.36, 132.9, 133.7, 133.9, 134.8, 137.2, 139.6, 140.2, 156.7, 156.9, 157.1, 157.7; The enantiomeric excess was determined by phenomenex cellulose-4 (25 cm), CH_3CN/IPA = 95/5, 0.5 mL/min, λ = 254 nm, *t* (major) = 9.818 min, *t* (minor) = 8.218 min.

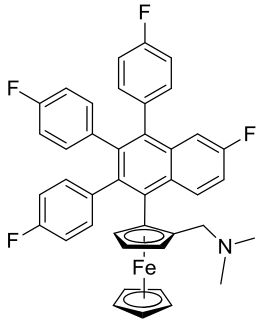

(S_P)-1-[(N,N-Dimethylamino)methyl]-2-(6-fluoro-2,3,4-tris(4-fluorophenyl)naphthalen-1-yl)ferrocene (**3ad**)

Yellow solid (41 mg, 31% yield, 97% ee). Analytical data for **3ad**: $[\alpha]_D^{20}$ = −62.2° (*c* = 0.30 chloroform, 97% ee). ^{1}H NMR (300 MHz, $CDCl_3$) δ 2.18 (s, 6H), 3.10 (d, *J* = 13.2 Hz, 1H), 3.44 (d, *J* = 13.5 Hz, 1H), 3.66 (s, 1H), 3.91 (t, *J* = 2.4 Hz, 1H), 4.18 (s, 5H), 4.45 (s, 1H), 6.39–6.66 (series of m, 7H), 6.79–6.86 (m, 3H), 7.08 (td, J_1 = 8.4 Hz, J_2 = 1.5 Hz, 1H), 7.18 (dd, J_1 = 11.1 Hz, J_2 = 2.7 Hz, 1H), 7.36 (td, J_1 = 6.6 Hz, J_2 = 2.1 Hz, 1H), 7.44 (td, J_1 = 8.4 Hz, J_2 = 2.4 Hz, 1H), 10.7 (dd, J_1 = 9.3 Hz, J_2 = 6.0 Hz, 1H); ^{13}C NMR (100 MHz, $CDCl_3$) δ 45.6, 57.9, 65.7, 69.6, 70.1, 71.8, 85.6, 86.9,

110.1 (d, *J* = 21.5 Hz), 113.6 (d, *J* = 21.2 Hz), 113.74 (d, *J* = 20.8 Hz), 113.78 (d, *J* = 20.9 Hz), 113.9 (d, *J* = 21.0 Hz), 114.2 (d, *J* = 26.7 Hz), 114.6 (d, *J* = 21.4 Hz), 115.2 (d, *J* = 20.6 Hz), 128.3, 131.7 (d, *J* = 8.1 Hz), 132.30, 132.38, 132.43, 132.50 (d, *J* = 8.7 Hz), 132.62, 133.0 (d, *J* = 7.7 Hz), 133.11, 133.6 (d, *J* = 8.0 Hz), 134.9 (d, *J* = 3.4 Hz), 136.2 (d, *J* = 3.1 Hz), 137.0 (d, *J* = 5.1 Hz), 137.5 (d, *J* = 3.7 Hz), 139.1, 140.6, 160.65 (d, *J* = 244 Hz), 160.74 (d, *J* = 246 Hz), 160.76 (d, *J* = 242 Hz), 161.6 (d, *J* = 245 Hz); ^{19}F NMR (376 MHz, $CDCl_3$) δ −116.8, −116.5, −115.2, −114.0; The enantiomeric excess was determined by Daicel Chiralcel OD-H (25 cm), hexanes/IPA = 98/2, 0.3 mL/min, λ = 254 nm, *t* (major) = 19.220 min, *t* (minor) = 16.953 min.

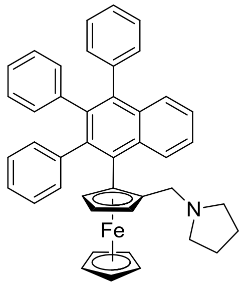

(*S*p)-1-[(Pyrrolidin-1-yl)-methyl]-2-(2,3,4-triphenylnaphthalen-1-yl)ferrocene (**3ba**) Yellow solid (44 mg, 35% yield, 95% ee). Analytical data for **3ba**: Mp = 77–79 °C; $[\alpha]_D^{20}$ = −101.4° (*c* = 0.31 chloroform, 95% ee). ^{1}H NMR (300 MHz, $CDCl_3$) δ 1.79 (br, 4H), 2.53 (br, 4H), 3.32 (d, *J* = 13.8 Hz, 1H), 3.71 (s, 1H), 3.76 (d, *J* = 13.5 Hz, 1H), 3.85 (s, 1H), 4.22 (s, 5H), 4.50 (s, 1H), 6.50 (d, *J* = 7.2 Hz, 1H), 6.63 (d, *J* = 6.0 Hz, 1H), 6.73–6.98 (series of m, 9H), 7.10 (t, *J* = 7.2 Hz, 1H), 7.18 (t, *J* = 7.5 Hz, 1H), 7.37 (t, *J* = 7.5 Hz, 1H), 7.42–7.51 (m, 2H), 7.63–7.70 (m, 2H), 10.22 (d, *J* = 9.0 Hz, 1H); ^{13}C NMR (100 MHz, $CDCl_3$) δ 23.4, 54.2, 54.5, 65.1, 68.8, 69.6, 70.0, 71.7, 85.8, 123.8, 125.1, 125.6, 126.21, 126.28, 126.38, 126.40, 126.5, 127.0, 127.1, 127.7, 129.0, 130.9, 131.17, 131.25, 131.36, 131.39, 131.8, 132.1, 132.2, 138.3, 138.8, 139.7, 140.7, 142.1 142.4; IR (film) 3445, 3055, 2959, 2775, 1651, 1601, 1491, 1441, 1373, 1107, 1031, 1002, 819, 754, 699 cm^{-1}; HRMS (ESI): Exact mass calcd for $C_{43}H_{38}FeN^{+1}$ (M+H) requires *m/z* 624.2348, found *m/z* 624.2339. The enantiomeric excess was determined by phenomenex cellulose-3 (25 cm), CH_3CN/IPA = 95 5, 0.5 mL/min, λ = 254 nm, *t* (major) = 6.917 min, *t* (minor) =7.473 min.

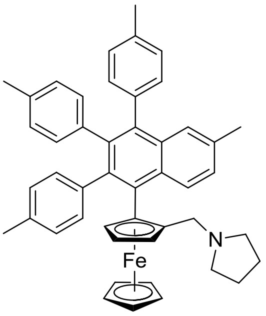

(S_P)-1-(Pyrrolidin-1-yl-methyl)-2-(6-methyl-2,3,4-tri-p-tolylnaphthalen-1-yl)ferrocene (**3bb**)

Yellow solid (41 mg, 30% yield, 97% ee). Analytical data for **3bb**: Mp = 84–86 ^oC; $[\alpha]_D^{20}$ = –157.7° (*c* = 0.31 chloroform, 97% ee). ^1H NMR (400 MHz, $CDCl_3$) δ 1.77 (br, 4H), 2.10 (s, 3H), 2.14 (s, 3H), 2.33 (s, 3H), 2.45 (s, 3H), 2.52 (br, 4H), 3.25 (d, *J* = 13.6 Hz, 1H), 3.67 (dd, J_1 = 2.0 Hz, J_2 = 1.2 Hz, 1H), 3.78 (d, *J* = 13.6 Hz, 1H), 3.82-3.83 (m, 1H), 4.18 (s, 5H), 4.49 (s, 1H), 6.36 (dd, J_1 = 8.0 Hz, J_2 = 1.6 Hz, 1H), 6.43–6.45 (m, 1H), 6.55 (d, *J* = 8.0 Hz, 1H), 6.61–6.65 (m, 3H), 6.69–6.73 (m, 2H), 6.81 (dd, J_1 = 8.0 Hz, J_2 = 1.6 Hz, 1H), 6.89 (d, *J* = 7.2 Hz, 1H), 7.17 (d, *J* = 7.6 Hz, 1H), 7.37 (dd, J_1 = 7.6 Hz, J_2 = 1.6 Hz, 1H), 7.38 (s, 1H), 7.48 (dd, J_1 = 8.8 Hz, J_2 = 1.6 Hz, 1H), 10.04 (d, *J* = 8.8 Hz, 1H); ^{13}C NMR (100 MHz, $CDCl_3$) δ 21.06, 21.09, 21.3, 21.8, 23.5, 54.3, 54.5, 65.0, 68.5, 69.9, 71.6, 86.2, 125.77, 125.83, 126.94, 127.01, 127.09, 127.11, 127.7, 128.4, 129.0, 129.5, 130.8, 131.0, 131.20, 131.23, 131.34, 131.67, 131.69, 132.5, 134.01, 134.11, 134.9, 135.3, 137.0, 137.7, 138.1, 139.0, 139.3, 141.7; IR (film) 3440, 3020, 2920, 2871, 2776, 1622, 1511, 1447, 1375, 1347, 1183, 1107, 833, 815, 731 cm^{-1}; HRMS (ESI): Exact mass calcd for $C_{47}H_{46}FeN^{+1}$ (M+H) requires *m/z* 680.2974, found *m/z* 680.2971. The enantiomeric excess was determined by phenomenex cellulose-4 (25 cm), CH_3CN/IPA = 95/5, 0.5 mL/min, λ = 254 nm, *t* (major) = 11.327 min, *t* (minor) = 9.835 min.

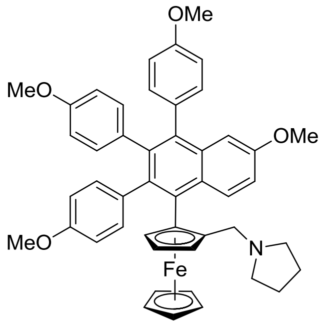

(S_P)-1-[(Pyrrolidin-1-yl)-methyl]-2-(6-methoxy-2,3,4-tris(4-methoxyphenyl)naphthale n-1-yl)ferrocene (**3bc**)

Yellow solid (61 mg, 41% yield, 97% ee). Analytical data for **3bc**: Mp = 87–89 °C; $[\alpha]_D^{20}$ = −174.3° (*c* = 0.30 chloroform, 97% ee). ^{1}H NMR (300 MHz, $CDCl_3$) δ 1.77 (br, 4H), 2.51 (br, 4H), 3.24 (d, *J* = 13.5 Hz, 1H), 3.62 (s, 3H), 3.66 (s, 3H), 3.74 (s, 3H), 3.79 (s, 3H), 3.69–3.89 (series of m, 3H), 4.17 (s, 5H), 4.47 (s, 1H), 6.31–6.47 (series of m, 6H), 6.60–6.65 (m, 2H), 6.75 (d, *J* = 8.7 Hz, 1H), 6.81 (d, *J* = 8.4 Hz, 1H), 6.91 (dd, J_1 = 8.4 Hz, J_2 = 2.4 Hz, 1H), 7.95 (d, *J* = 2.4 Hz, 1H), 7.30 (dd, J_1 = 9.3 Hz, J_2 = 2.4 Hz, 1H), 7.38 (d, *J* = 8.4 Hz, 1H), 10.11 (d, *J* = 9.3 Hz, 1H); ^{13}C NMR (75 MHz, $CDCl_3$) δ 23.4, 54.1, 54.5, 54.78, 54.89, 54.97, 55.02, 65.1, 68.5, 69.4, 69.8, 71.5, 86.1, 87.8, 105.5, 111.8, 112.0, 112.6, 113.2, 115.4, 126.7, 130.8, 131.9, 132.11, 132.18, 132.31, 132.34, 132.7, 133.6, 133.8, 134.8, 137.0, 139.4, 140.1, 156.6, 156.8, 157.0, 157.6; IR (film) 3423, 2932, 2833, 2776, 1609, 1509, 1459, 1286, 1245, 1176, 1107, 1035, 827, 774 cm^{-1}; HRMS (ESI): Exact mass calcd for $C_{47}H_{46}FeNO_4^{+1}$ (M+H) requires *m/z* 744.2771, found *m/z* 744.2770. The enantiomeric excess was determined by phenomenex cellulose-4 (25 cm), CH_3CN/IPA = 95/5, 0.5 mL/min, λ = 254 nm, *t* (major) = 11.677 min, *t* (minor) = 8.627 min.

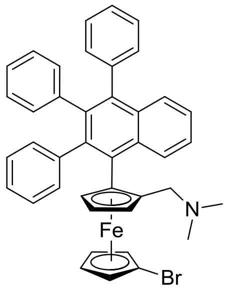

(S_P)-1-[(N,N-Dimethylamino)methyl]-2-(2,3,4-triphenylnaphthalen-1-yl)-1'-bromoferrocene (**3ca**)

Yellow solid (85 mg, 42% yield, 96% ee). Analytical data for **3ca**: Mp = 82–84 °C; $[\alpha]_D^{20}$ = −13.3° (*c* = 0.15 chloroform, 96% ee). ^{1}H NMR (400 MHz, $CDCl_3$) δ 2.31 (s, 6H), 3.12 (d, *J* = 14.0 Hz, 1H), 3.79 (d, *J* = 14.0 Hz, 1H), 3.89 (s, 1H), 3.94 (t, *J* = 2.4 Hz, 1H), 3.86 (d, *J* = 1.2 Hz, 1H), 4.23 (d, *J* = 0.8 Hz, 1H), 4.47 (d, *J* = 1.2 Hz, 1H), 4.49 (d, *J* = 0.8 Hz, 1H), 4.67 (s, 1H), 6.61 (d, *J* = 7.6 Hz, 1H), 6.74 (t, *J* = 2.8 Hz, 1H), 6.80–6.98 (series of m, 8H), 7.05 (d, *J* = 8.0 Hz, 1H), 7.14 (t, *J* = 7.6 Hz, 1H), 7.23 (t, *J* = 7.6 Hz, 1H), 7.42 (t, *J* = 7.6 Hz, 1H), 7.51 (t, *J* = 7.6 Hz, 1H), 7.56 (d, *J* = 8.0 Hz, 1H), 7.74 (d, *J* = 8.0 Hz, 1H), 7.80 (t, *J* = 7.6 Hz, 1H), 10.01 (d, *J* = 8.4 Hz, 1H); ^{13}C NMR (100 MHz, $CDCl_3$) δ 46.0, 56.6, 68.4, 68.6, 69.5, 70.3, 71.7, 73.4, 74.3, 79.6, 87.6, 88.6, 124.2, 125.28, 125.39, 125.9, 126.46, 126.54, 126.58, 126.74, 127.2, 127.9, 128.9, 131.0, 131.23, 131.25, 131.35, 131.45, 131.93, 132.23, 138.7, 139.0, 139.7, 140.8, 142.1, 142.6; IR (film) 3056, 2941, 2814, 2765, 1602, 1491, 1442, 1370, 1265, 1152, 1072, 1024, 872, 789, 772, 755, 737, 700, 624 cm^{-1}; HRMS (ESI): Exact mass calcd for $C_{41}H_{35}BrFeN^{+1}$ (M+H) requires *m/z* 676.1297, found *m/z* 676.1287. The enantiomeric excess was determined by Daicel Chiralcel OD-H (25 cm), hexanes/IPA = 98/2, 0.4 mL/min, λ = 254 nm, *t* (major) = 16.438 min, *t* (minor) = 14.545 min.

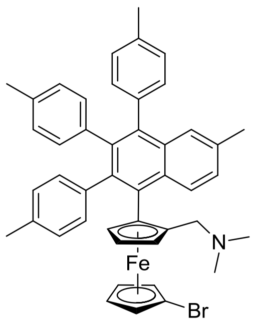

(S_P)-1-[(N,N-Dimethylamino)methyl]-2-(6-methyl-2,3,4-tri-*p*-tolylnaphthalen-1-yl)-1’ -bromo ferrocene (**3cb**)

Yellow solid (41 mg, 28% yield, 96% ee). Analytical data for **3cb**: Mp = 87–89 °C; $[\alpha]_D^{20}$ = −59.7° (*c* = 0.29 chloroform, 96% ee). ^{1}H NMR (400 MHz, $CDCl_3$) δ 2.09 (s, 3H), 2.15 (s, 3H), 2.25 (s, 6H), 2.32 (s, 3H), 2.45 (s, 3H), 3.00 (d, *J* = 14.0 Hz, 1H), 3.77–3.80 (m, 2H), 3.85 (t, *J* = 2.8 Hz, 1H), 4.00 (dd, J_1 = 4.0 Hz, J_2 = 2.4 Hz, 1H), 4.15 (dd, J_1 = 4.0 Hz, J_2 = 2.4 Hz, 1H), 4.38–4.39 (m, 2H), 4.64 (br, 1H), 6.35 (dd, J_1 = 7.6 Hz, J_2 = 1.6 Hz, 1H), 6.41 (d, *J* = 7.2 Hz, 1H), 6.55 (d, *J* = 8.0 Hz, 1H), 6.62-6.74 (series of m, 5H), 6.80 (dd, J_1 = 8.0 Hz, J_2 = 1.6 Hz, 1H), 6.89 (dt, J_1 = 8.0 Hz, J_2 = 0.8 Hz, 1H), 7.16 (d, *J* = 7.6 Hz, 1H), 7.32 (dd, J_1 = 8.0 Hz, J_2 = 1.6 Hz, 1H), 7.38 (s, 1H), 7.52 (dd, J_1 = 8.4 Hz, J_2 = 1.6 Hz, 1H), 9.73 (d, *J* = 8.8 Hz, 1H); ^{13}C NMR (100 MHz, $CDCl_3$) δ 21.00, 21.04, 21.21, 21.7, 45.5, 56.4, 68.2, 68.6, 69.3, 70.2, 71.3, 73.2, 74.3, 79.5, 87.8, 126.0, 126.1, 127.00, 127.08, 127.10, 127.12, 127.7, 128.4, 128.6, 129.2, 130.69, 130.92, 130.99, 131.16, 131.24, 131.7, 132.4, 134.1, 134.4, 135.0, 135.4, 136.8, 137.9, 138.1, 139.07, 139.16, 141.8; IR (film) 3421, 3021, 2920, 1814, 2761, 2602, 2496, 1508, 1151, 1107, 1022, 869, 834, 815, 742, 668 cm^{-1}; HRMS (ESI): Exact mass calcd for $C_{45}H_{43}BrFeN^{+1}$ (M+H) requires *m/z* 732.1923, found *m/z* 732.1919. The enantiomeric excess was determined by phenomenex cellulose-4 (25 cm), CH_3CN/IPA = 95/5, 0.5 mL/min, λ = 254 nm, *t* (major) = 17.027 min, *t* (minor) = 16.227 min.

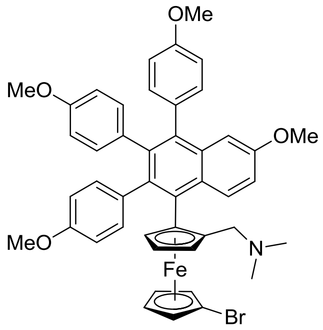

(S_P)-1-[(N,N-Dimethylamino)methyl]-2-(6-methoxy-2,3,4-tris(4-methoxyphenyl)naphthalen-1-yl)-1'-bromo ferrocene (**3cc**)

Yellow solid (64 mg, 40% yield, 96% ee). Analytical data for **3cc**: Mp = 90–92 °C; $[\alpha]_D^{20}$ = −80.7° (*c* = 0.30 chloroform, 96% ee). ^{1}H NMR (400 MHz, $CDCl_3$) δ 2.22 (s, 6H), 2.98 (d, *J* = 13.6 Hz, 1H), 3.62 (s, 3H), 3.66 (s, 3H), 3.68 (d, *J* = 18.4 Hz, 1H), 3.75 (s, 3H), 3.76 (dd, J_1 = 2.4 Hz, J_2 = 1.6 Hz, 1H), 3.79 (s, 3H), 3.88 (t, *J* = 2.8 Hz, 1H), 4.00 (td, J_1 = 2.8 Hz, J_2 = 1.2 Hz, 1H), 4.14 (td, J_1 = 2.8 Hz, J_2 = 1.2 Hz, 1H), 4.36–4.38 (m, 2H), 4.58 (s, 1H), 6.31–6.48 (series of m, 6H), 6.63 (t, *J* = 2.4 Hz, 1H), 6.65 (t, *J* = 2.0 Hz, 1H), 6.76 (dd, J_1 = 8.4 Hz, J_2 = 2.4 Hz, 1H), 6.81 (dd, J_1 = 8.4 Hz, J_2 = 2.4 Hz, 1H), 6.90 (dd, J_1 = 8.4 Hz, J_2 = 2.4 Hz, 1H), 7.96 (d, *J* = 2.8 Hz, 1H), 7.34–7.38 (m, 2H), 9.80 (d, *J* = 9.6 Hz, 1H); ^{13}C NMR (100 MHz, $CDCl_3$) δ 45.6, 54.84, 54.96, 55.03, 55.11, 56.4, 68.2, 68.7, 69.3, 70.2, 71.4, 73.1, 74.2, 79.4, 87.8, 105.7, 111.9, 112.1, 112.7, 113.3, 115.8, 126.5, 130.5, 131.1, 131.96, 132.10, 132.12, 132.21, 132.28, 132.9, 133.6, 133.8, 134.7, 137.4, 139.6, 140.3, 156.7, 157.0, 157.2, 157.7; IR (film) 3384, 2934, 2833, 2762, 1735, 1610, 1577, 1509, 1459, 1370, 1286, 1286, 1244, 1176, 1035, 828 cm^{-1}; HRMS (ESI): Exact mass calcd for $C_{45}H_{43}BrFeNO_4^{+1}$ (M+H) requires *m/z* 796.1719, found *m/z* 796.1714. The enantiomeric excess was determined by phenomenex cellulose-4 (25 cm), CH_3CN/IPA = 95/5, 0.5 mL/min, λ = 254 nm, *t* (major) = 9.277 min, *t* (minor) = 8.377 min.

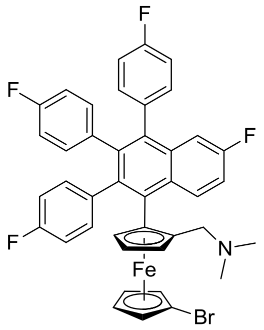

(S_P)-1-[(N,N-Dimethylamino)methyl]-2-(6-methoxy-2,3,4-tris(4-methoxyphenyl)naphthalen-1-yl)ferrocene (**3cd**)

Yellow solid (45 mg, 30% yield, 92% ee). Analytical data for **3cd**: Mp = 94–96 °C; $[\alpha]_D^{20}$ = −9.7° (*c* = 0.31 chloroform, 92% ee). ^{1}H NMR (300 MHz, $CDCl_3$) δ 2.27 (s, 6H), 3.11 (d, *J* = 13.5 Hz, 1H), 3.71–3.75 (m, 2H), 3.97 (br, 1H), 4.05 (br, 1H), 4.18 (br, 1H), 4.35 (d, *J* = 0.9 Hz, 1H), 4.44 (br, 1H), 4.68 (br, 1H), 6.43–6.71 (series of m, 7H), 6.80-6.86 (m, 3H), 7.08 (td, J_1 = 8.1 Hz, J_2 = 1.2 Hz, 1H), 7.20 (dd, J_1 = 10.8 Hz, J_2 = 2.7 Hz, 1H), 7.34-7.39 (td, J_1 = 6.9 Hz, J_2 = 1.5 Hz,1H), 7.48-7.55 (td, J_1 = 8.4 Hz, J_2 = 2.7 Hz, 1H), 9.98 (dd, J_1 = 9.3 Hz, J_2 = 6.0 Hz, 1H); ^{13}C NMR (100 MHz, $CDCl_3$) δ 45.68, 45.80, 56.3, 68.3, 68.8, 69.2, 70.1, 70.5, 72.0, 73.3, 74.1, 79.4, 87.2, 110.1 (d, *J* = 21.8 Hz), 113.7 (d, *J* = 21.4 Hz), 113.8 (d, *J* = 21.2 Hz), 113.9 (d, *J* = 21.3 Hz), 114.5 (d, *J* = 24.8 Hz), 114.6 (d, *J* = 21.5 Hz), 115.2 (d, *J* = 21.2 Hz), 128.1, 131.5 (d, *J* = 8.3 Hz), 132.28, 132.33, 132.37, 132.45, 132.52, 132.6 (d, *J* = 7.8 Hz), 133.0 (d, *J* = 7.7 Hz), 133.6 (d, *J* = 8.2 Hz), 134.8 (d, *J* = 3.4 Hz), 136.1 (d, *J* = 3.3 Hz), 137.3, 137.4 (d, *J* = 3.1 Hz), 139.2, 140.7, 160.7 (d, *J* = 244 Hz), 160.815 (d, *J* = 247 Hz), 160.824 (d, *J* = 244 Hz), 161.6 (d, *J* = 245 Hz); ^{19}F NMR (376 MHz, $CDCl_3$) δ -116.2, -116.1, -115.0, -113.3; IR (film) 2941, 2814, 2765, 2604, 2498, 1625, 1604, 1513, 1368, 1228, 1156, 1093, 1039, 1015, 872, 847, 831, 819, 785 cm^{-1}; HRMS (ESI): Exact mass calcd for $C_{41}H_{31}BrF_4FeN^{+1}$ (M+H) requires *m/z* 748.0920, found *m/z* 748.0920. The enantiomeric excess was determined by Daicel Chiralcel OD-H (25 cm), hexanes/IPA = 98/2, 0.3 mL/min, λ = 254 nm, *t* (major) = 20.938 min, *t* (minor) = 17.655 min.

Synthesis of (S_p)-L1

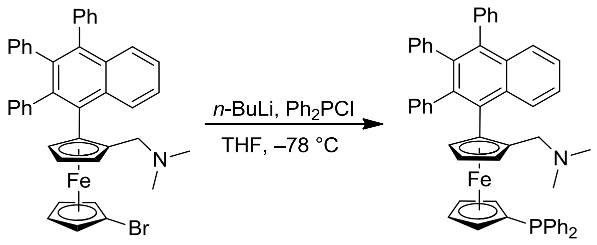

(S_p)-**3ca**, 96% ee (S_p)-**L1**, 43% yield, 97% ee

To a solution of compound (S_p)-**3ca** (610 mg, 0.90 mmol) in THF (10 mL) was added *n*-BuLi (0.68 mL, 1.08 mmol, 1.6 M in *n*-hexane) at −78 °C under argon. The resulting deep red solution was stirred for 30 min. Then chlorodiphenylphosphine (0.194 mL, 1.08 mmol) was added. The mixture was warmed slowly to 0 °C and stirred for 1 h. Then the reaction mixture was quenched with water, extracted with ethyl acetate. The combined organic layers were washed with brine, dried over Na_2SO_4, and filtrated. After the solvent was removed under reduced pressure, the residue was purified by silica gel column chromatography (dichloromethane/methanol = 1/100, v/v) to give (S_p)-**L1** (306 mg, 43% yield, 97% ee) as an orange solid. Analytical data for (S_p)-**L1**: Mp = 93–95 °C; $[\alpha]_D^{20}$ = −130.6° (*c* = 0.63 chloroform, 97% ee). ^{1}H NMR (400 MHz, $CDCl_3$) δ 2.18 (s, 6H), 3.11 (d, *J* = 13.6 Hz, 1H), 3.62 (d, *J* = 13.6 Hz, 1H), 3.76 (br, 1H), 3.78 (t, *J* = 2.4 Hz, 1H), 4.04 (s, 1H), 4.13 (br, 1H), 4.39 (br, 1H), 4.48 (br, 1H), 4.52 (br, 1H), 6.47 (d, *J* = 7.6 Hz, 1H), 6.53 (d, *J* = 7.2 Hz, 1H), 6.72 (td, J_1 = 7.6 Hz, J_2 = 0.8 Hz, 1H), 6.76–6.90 (series of m, 7H), 6.96 (d, *J* = 7.6 Hz, 1H), 7.02–7.08 (m, 3H), 7.11–7.31 (series of m, 9H), 7.33–7.38 (m, 2H), 7.48 (d, *J* = 7.6 Hz, 1H), 7.534 (td, J_1 = 9.4 Hz, J_2 = 1.2 Hz, 1H), 7.59 (d, *J* = 8.4 Hz, 1H), 9.86 (d, *J* = 8.4 Hz, 1H); ^{13}C NMR (100 MHz, $CDCl_3$) δ 45.2, 57.8, 66.8, 70.6, 73.3, 73.51, 73.58, 73.62, 74.07, 74.10, 74.19, 74.95, 75.07, 76.55, 76.61, 86.7, 123.8, 125.17, 125.32, 123.6, 126.36, 126.42, 126.44, 126.46, 126.6, 127.09, 127.16, 127.74, 127.79, 127.80, 128.10, 128.17, 128.24, 128.56, 128.75, 130.97, 131.00, 131.18, 131.27, 131.33, 132.0, 132.2, 133.3 (d, *J* = 18.6 Hz), 133.5 (d, *J* = 19.2 Hz), 138.45, 138.54, 138.74, 138.83, 138.94, 139.7, 140.7, 142.0, 142.4; ^{31}P NMR (161 MHz, $CDCl_3$) δ −18.05; IR (film) 3054, 2941, 2854, 2813, 2765, 1737, 1601, 1434, 1370, 1265, 1160, 1071, 1027, 846, 789, 772, 739, 698 cm^{-1}; HRMS (ESI): Exact mass

calcd for $C_{53}H_{45}FeNP^{+1}$ (M+H) requires *m/z* 782.2634, found *m/z* 782.2643. The enantiomeric excess was determined by phenomenex cellulose-2 (25 cm), hexanes/EtOH = 94/6, 0.7 mL/min, λ = 254 nm, *t* (major) = 8.690 min, *t* (minor) = 7.317 min.

Pd-catalyzed allylic amination reaction with (S_p)-**L1**

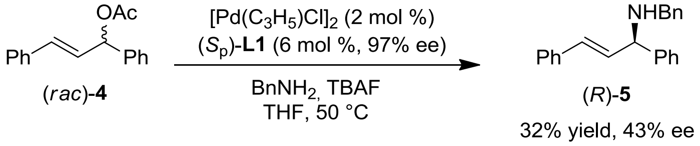

$[Pd(C_3H_5)Cl]_2$ (1.5 mg, 0.004 mmol) and ligand (S_p)-**L1** (9.4 mg, 0.012 mmol, 99% ee) were dissolved in dry THF (0.5 mL), and the mixture was stirred for 30 min at rt under argon. To this solution were successively added (*rac*)-**4** (50.4 mg, 0.2 mmol), $BnNH_2$ (42.8 mg, 0.4 mmol), and TBAF (1 M in THF, 0.4 mL, 0.4 mmol). The reaction mixture was stirred at 50 °C for 14 h. After completion, the reaction mixture was diluted with Et_2O (20 mL) and washed twice with ice-cold saturated aqueous ammonium chloride. The organic phase was dried over anhydrous Na_2SO_4 and then concentrated under reduced pressure. The residue was purified by preparative TLC (ethyl acetate/petroleum = 1/10) to give (*R*)-**5** (19.1 mg, 32% yield, 43% ee). Analytical data for (*R*)-**5**: $[\alpha]_D^{20}$ = −14.24° (*c* = 0.25 chloroform, 43% ee). ^{1}H NMR (300 MHz, $CDCl_3$) δ 1.84 (s, 1H), 3.78 (s, 2H), 4.39 (d, *J* = 6.9 Hz, 1H), 6.31 (dd, J_1 = 15.9 Hz, J_2 = 7.2 Hz, 1H), 6.57 (d, *J* = 15.9 Hz , 1H) 7.20–7.42 (series of m, 15H); The enantiomeric excess was determined by Diacel Chiralcel OJ-H (25 cm), hexanes/IPA = 90/10, 0.6 mL/min, λ = 254 nm, *t* (minor) = 18.40 min, *t* (major) = 22.07 min. The absolute configuration of the product (*R*)-**5** was assigned as (*R*) by comparing the optical rotation with that reported in the literature [5].

Pd-catalyzed allylic alkylation reaction with (S_p)-**L1**

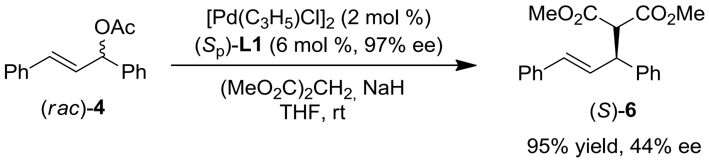

A mixture of ligand (S_p)-**L1** (9.4 mg, 0.012 mmol, 97% ee) and $[Pd(C_3H_5)Cl]_2$ (1.5 mg, 0.004 mmol) in dry THF (2 mL) was stirred at room temperature for 0.5 h, and to the resulting yellow solution was added **4** (50.4 mg, 0.2 mmol). After an additional stirring for 10 min, sodium dimethyl malonate [generated in situ by adding dimethyl malonate (79.2 mg, 0.6 mmol) and sodium hydride (14.4 mg, 0.6 mmol) in 1 mL THF] was added. The reaction was stirred at room temperature. After completion of the reaction (monitored by TLC), the reaction mixture was quenched with NH_4Cl (aq.) and extracted with ether. The combined organic layers were washed with brine, dried over anhydrous Na_2SO_4 and filtrated. After the solvent was removed under reduced pressure, the residue was purified by silica gel column chromatography (ethyl acetate/petroleum = 1/15) to give (*S*)-**6** (61 mg, 94% yield, 44% ee). Analytical data for (*S*)-**6**[6]: $[\alpha]_D^{20}$ = −8.8° (*c* = 1.4 chloroform, 44% ee). ^{1}H NMR (300 MHz, $CDCl_3$) δ 3.52 (s, 3H), 3.70 (s, 3H), 3.96 (d, *J* = 10.8 Hz, 1H), 4.27 (dd, *J* = 8.8, 10.4 Hz , 1H), 6.33 (dd, *J* = 8.4, 15.9 Hz , 1H), 6.48 (d, *J* = 15.6 Hz, 1H), 7.17–7.33 (series of m, 10H); The enantiomeric excess was determined by Diacel Chiralcel OD-H (25 cm), hexanes/IPA = 90/10, 0.7 mL/min, λ = 254 nm, *t* (minor) = 8.507 min, *t* (major) = 9.140 min. The absolute configuration of the product (*S*)-**6** was assigned as (*S*) by comparing the optical rotation with that reported in the literature [6].

References

(1) Khrushcheva, N. S.; Sokolov, V. I. *Russ. Chem. Bull.* **2004,** *53,* 830–833.

(2) Bhat, A. R.; Bhat, A. I.; Athar, F.; Azam, A. *Helv. Chim. Act.* **2009,** *92,* 1644–1656.

(3) Michael, W.; Ulrike, N.; Kurt, M. *Tetrahedron: Asymmetry* **1999,** *10,* 4369–4391.

(4) Mio, M. J.; Kopel, L. C.; Braun, J. B.; Gadzikwa, T. L.; Hull, K. L.; Brisbois, R. G. ; Markworth, C. J.; Grieco, P. A. *Org. Lett.* **2002,** *4,* 3199–3202

(5) Hayashi, T.; Yamamoto, A.; Ito, Y.; Nishioka, E.; Miura, H.; Yanagi, K. *J. Am. Chem. Soc.* **1989,** *111,* 6301–6311.

(6) Hayashi, T.; Yamamoto, A.; Hagihara, T.; Ito, Y. *Tetrahedron Lett*. **1986,** *27,* 191–194.

Copies of NMR spectra and HPLC chromatographs

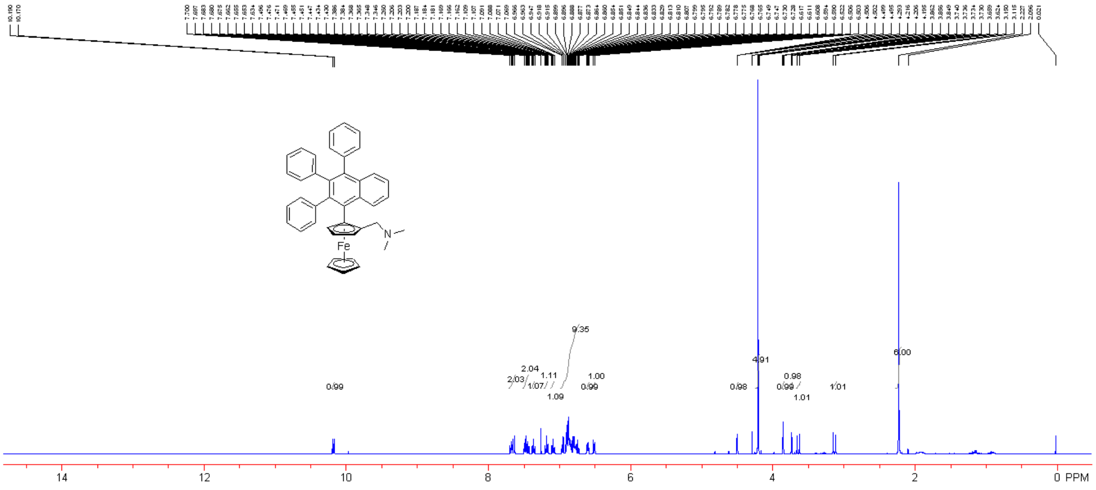

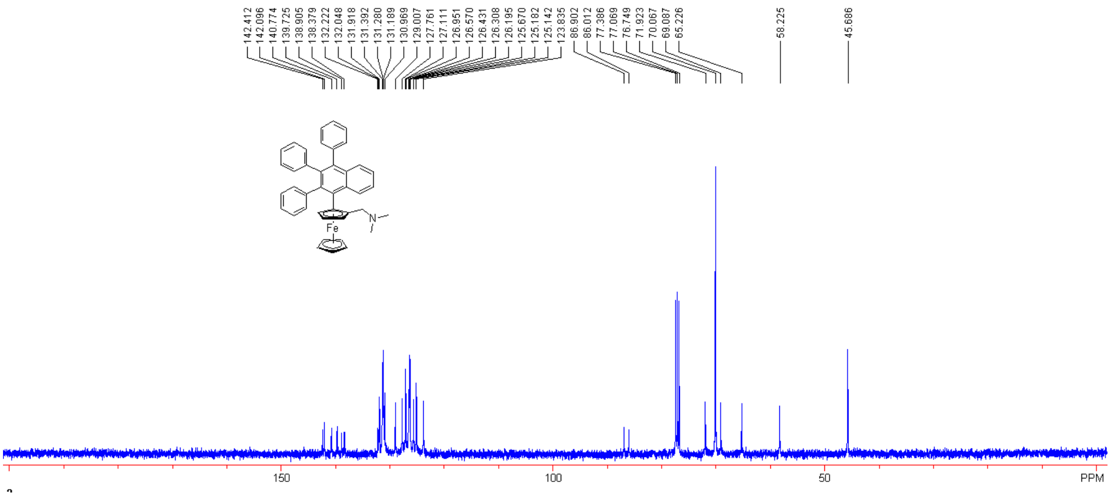

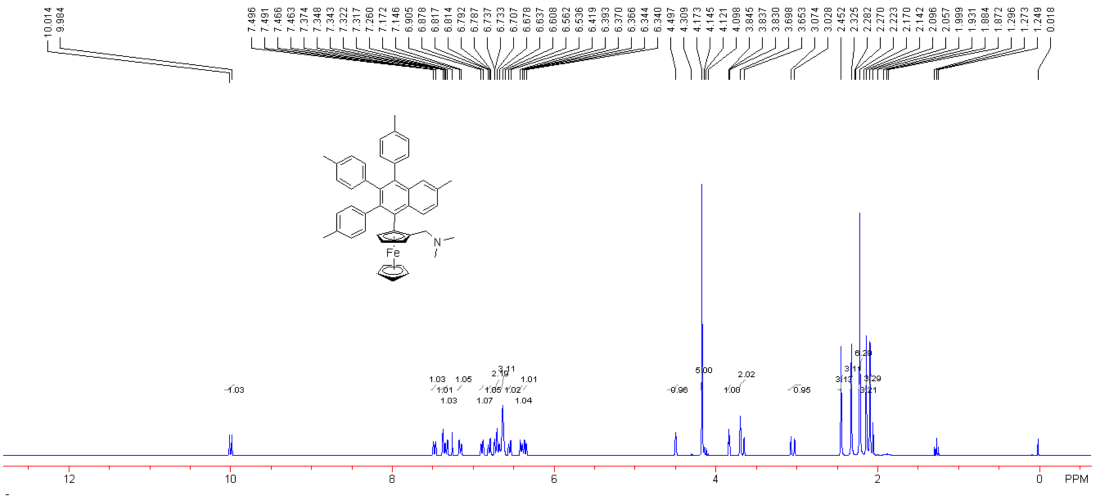

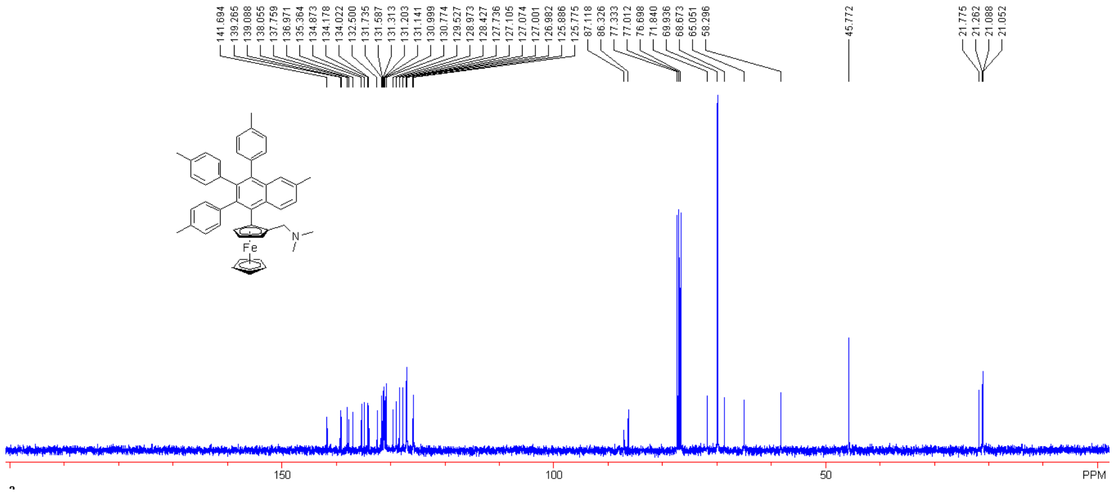
141.694
139.265
139.088
138.055
137.759
136.971
135.364
134.873
134.178
134.022
132.500
131.735
131.587
131.313
131.203
131.141
130.999
130.774
129.527
128.973
128.427
127.736
127.105
127.074
127.001
126.982
125.886
125.775
87.118
86.326
77.333
77.012
76.698
71.840
69.936
68.673
65.051
58.296
45.772
21.775
21.262
21.088
21.052
Fe
N
150
100
50
PPM

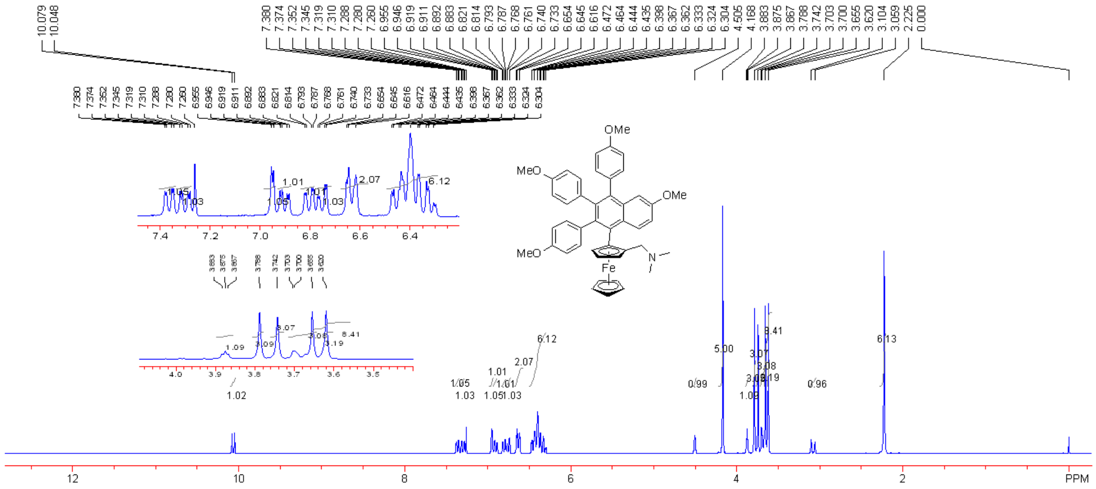
10.079
10.048
7.380
7.374
7.352
7.345
7.319
7.310
7.288
7.280
7.260
6.955
6.946
6.919
6.911
6.892
6.883
6.821
6.814
6.793
6.787
6.768
6.761
6.740
6.733
6.654
6.645
6.616
6.472
6.464
6.444
6.435
6.398
6.367
6.362
6.333
6.324
6.304
4.505
4.168
3.883
3.875
3.867
3.788
3.742
3.703
3.700
3.655
3.620
3.104
3.059
2.225
0.000
OMe
MeO
OMe
MeO
N
Fe
1.01
2.07
6.12
1.03
7.4
7.2
7.0
6.8
6.6
6.4
1.09
3.09
3.07
3.06
3.19
8.41
1.02
4.0
3.9
3.8
3.7
3.6
3.5
6.12
2.07
1.01
1.05
1.03
1.05
1.01
1.03
5.00
3.01
3.08
0.99
3.19
1.02
0.96
6.13
12
10
8
6
4
2
PPM

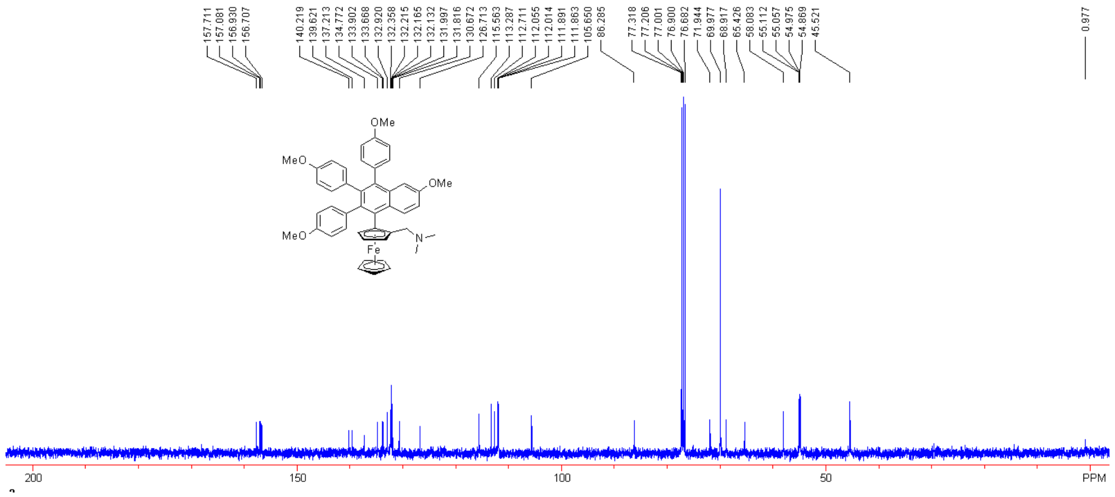
157.711
157.081
156.930
156.707
140.219
139.621
137.213
134.772
133.902
133.668
132.920
132.358
132.215
132.165
132.132
131.997
131.816
130.713
130.672
126.713
115.563
113.287
112.711
112.055
112.014
111.891
111.863
105.650
86.285
77.318
77.206
77.001
76.900
76.682
71.944
69.977
68.917
65.426
58.083
55.112
55.057
54.975
54.869
45.521
0.977
OMe
MeO
OMe
MeO
N
Fe
200
150
100
50
PPM

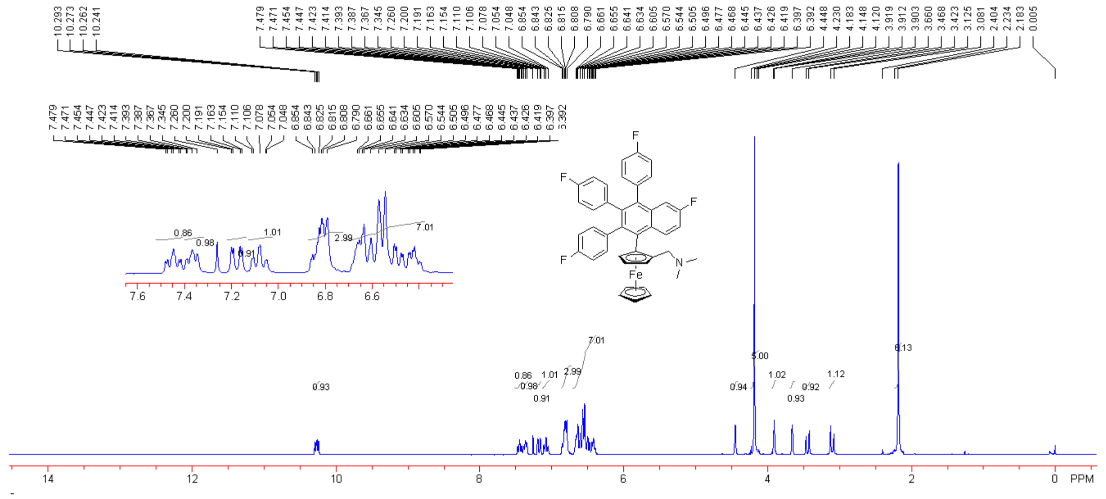

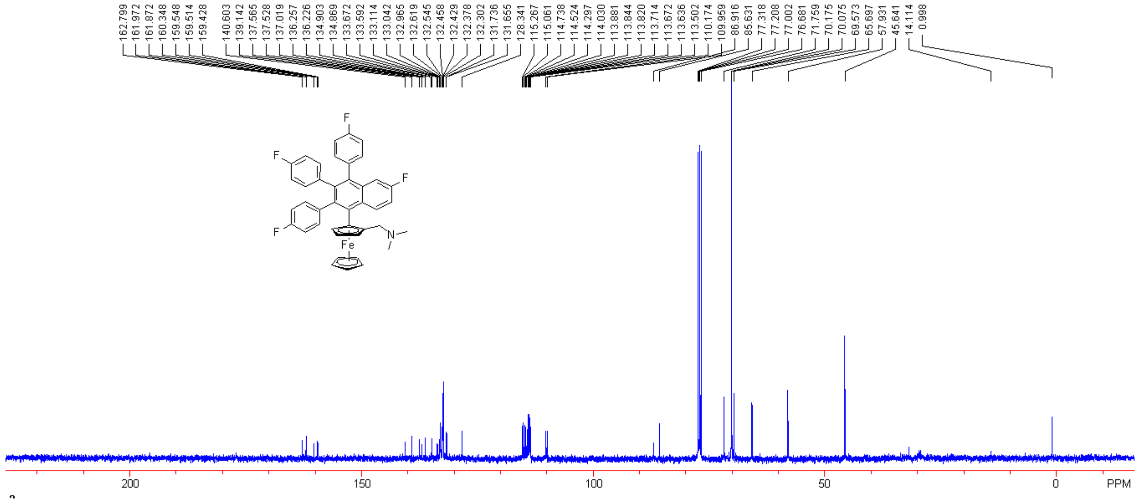

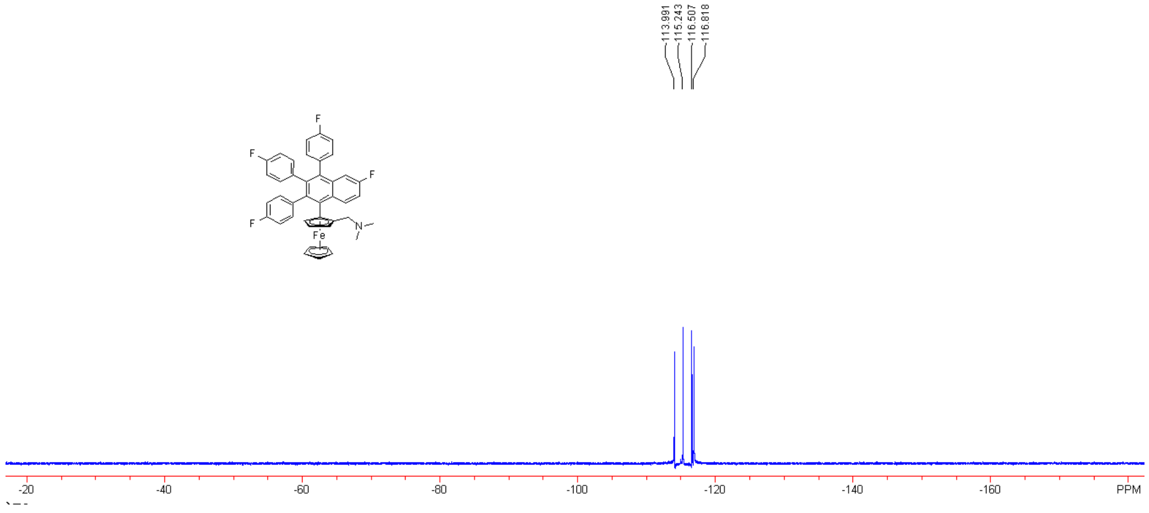

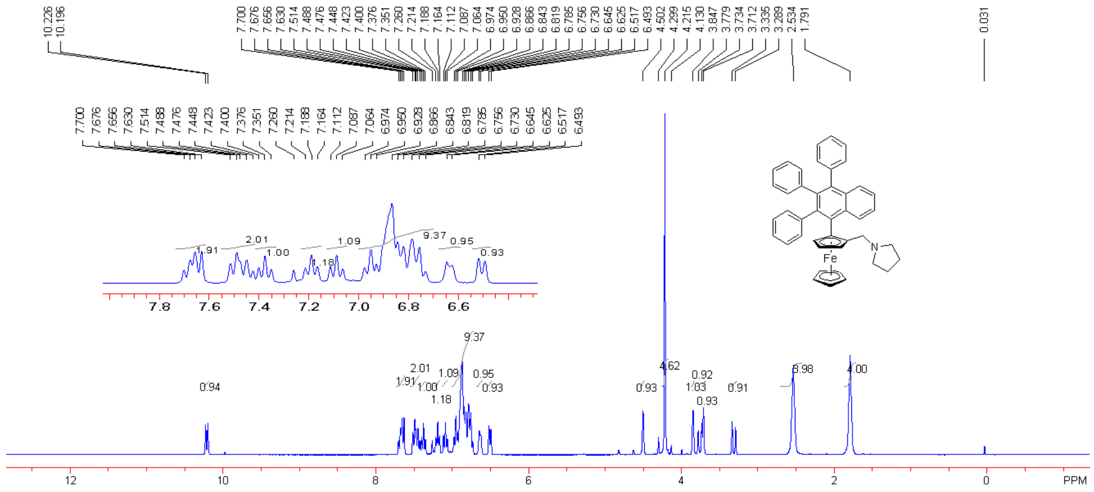

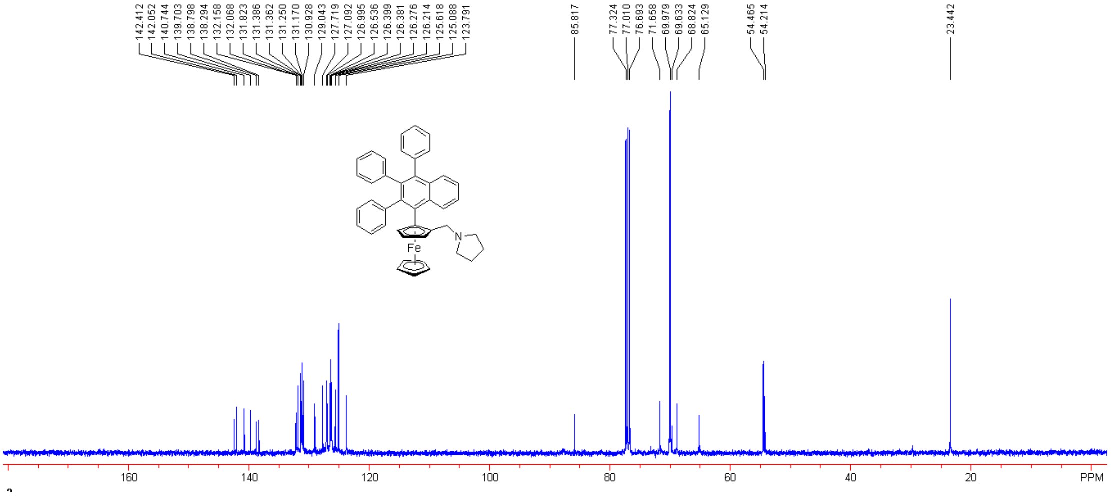

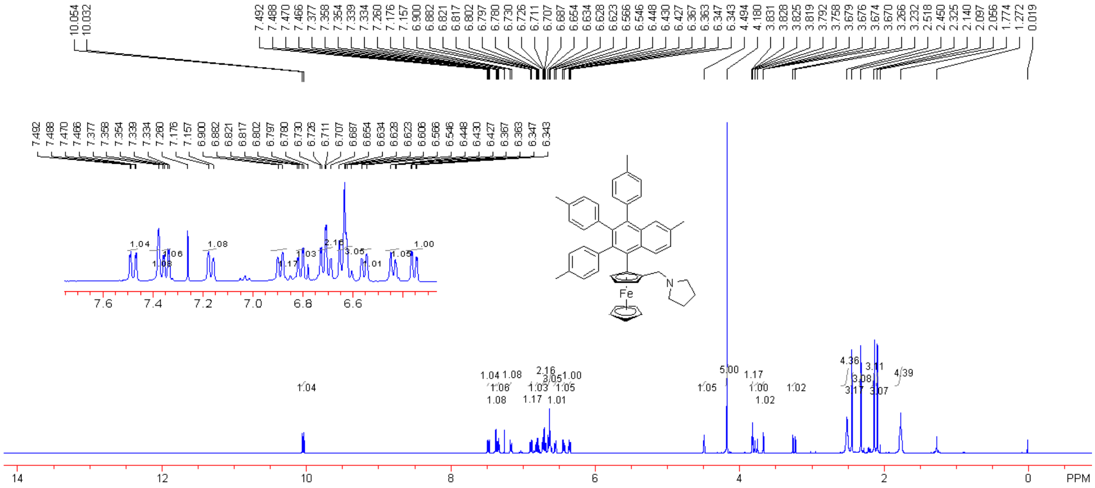

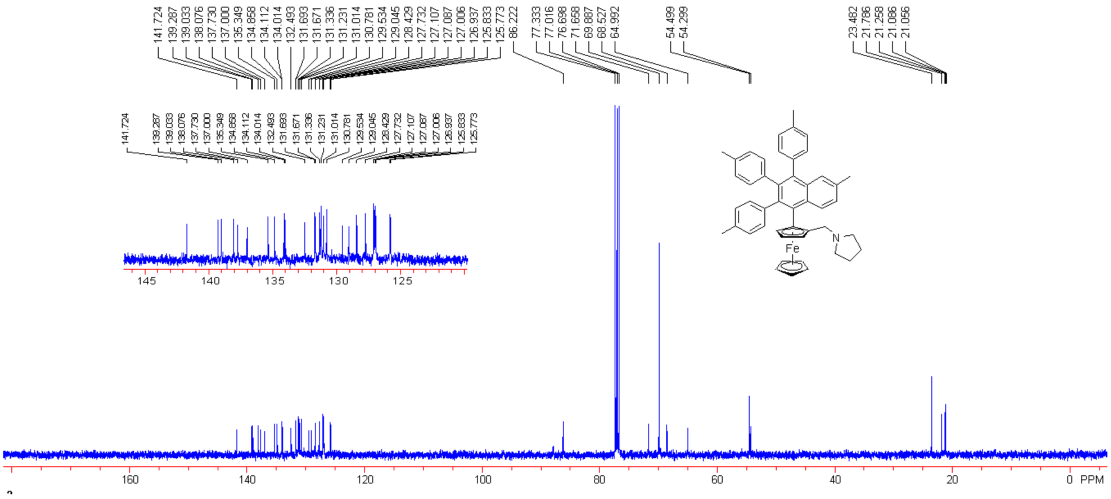

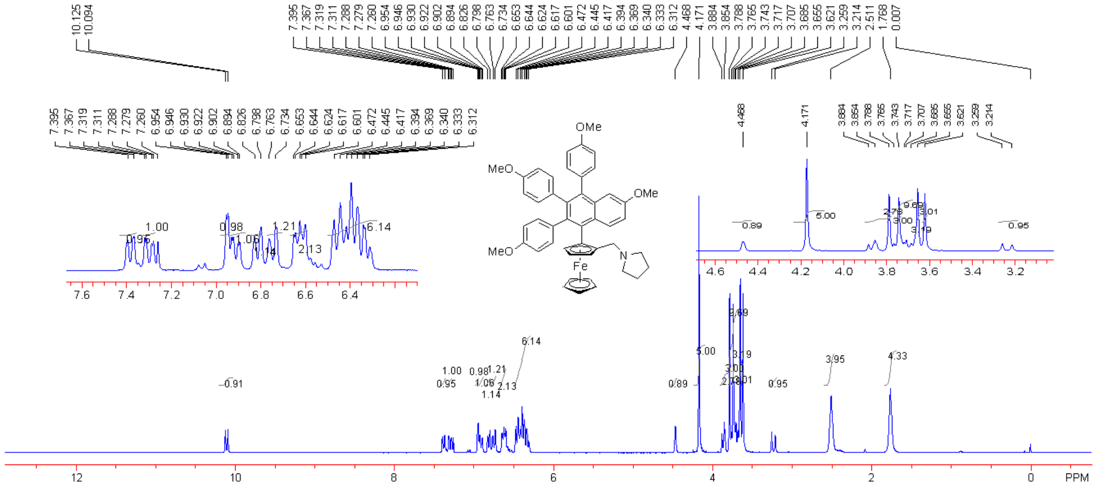

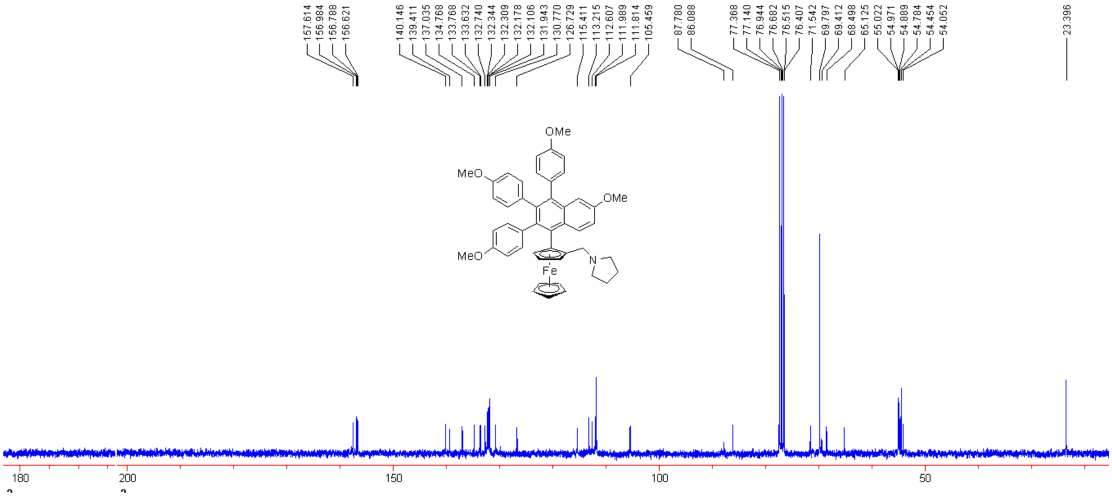

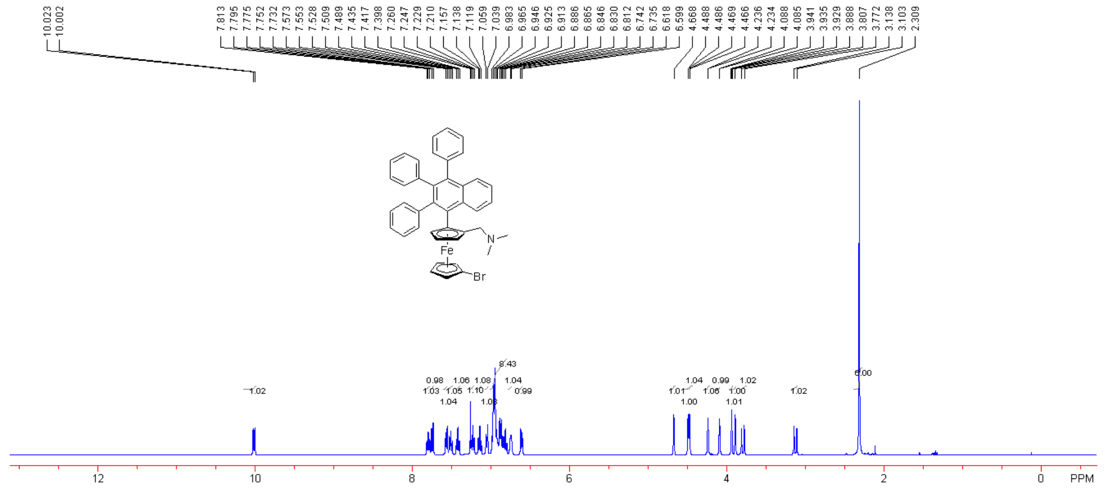
Fe
Br
N
PPM

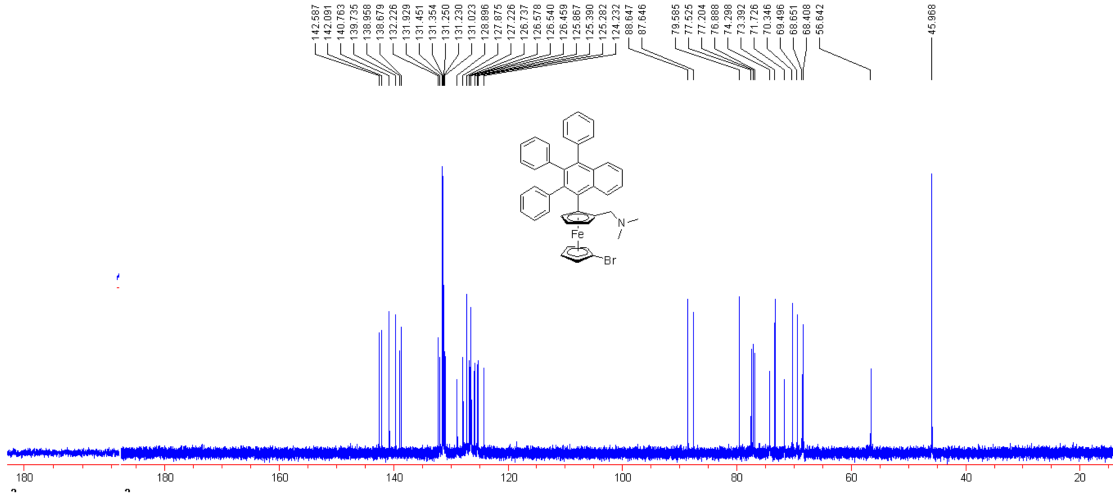
Fe
Br
N

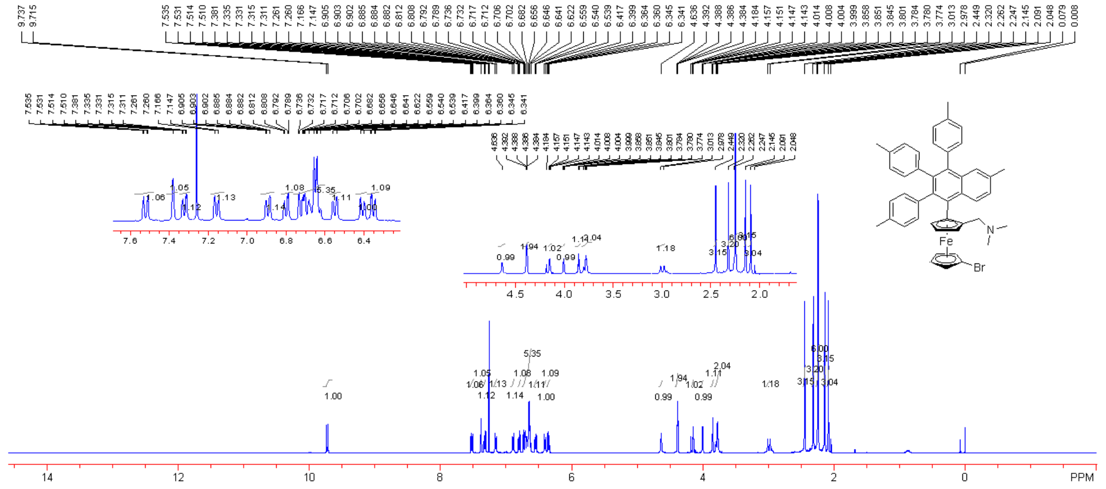
Fe
Br
N
PPM

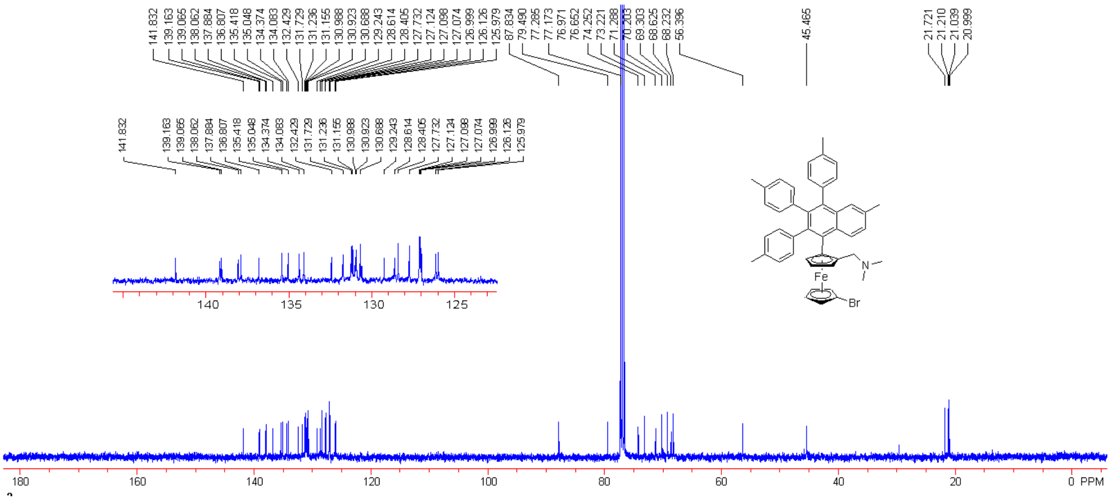
141.832
139.163
139.065
138.062
137.884
136.807
135.418
135.048
134.374
134.083
132.429
131.729
131.236
131.155
130.988
130.923
130.688
129.243
128.614
128.405
127.732
127.124
127.098
127.074
126.999
126.126
125.979
87.834
79.490
77.285
77.173
76.971
76.652
74.252
73.221
71.288
70.203
69.303
68.625
68.232
56.396
45.465
21.721
21.210
21.039
20.999
Fe
Br
N
PPM

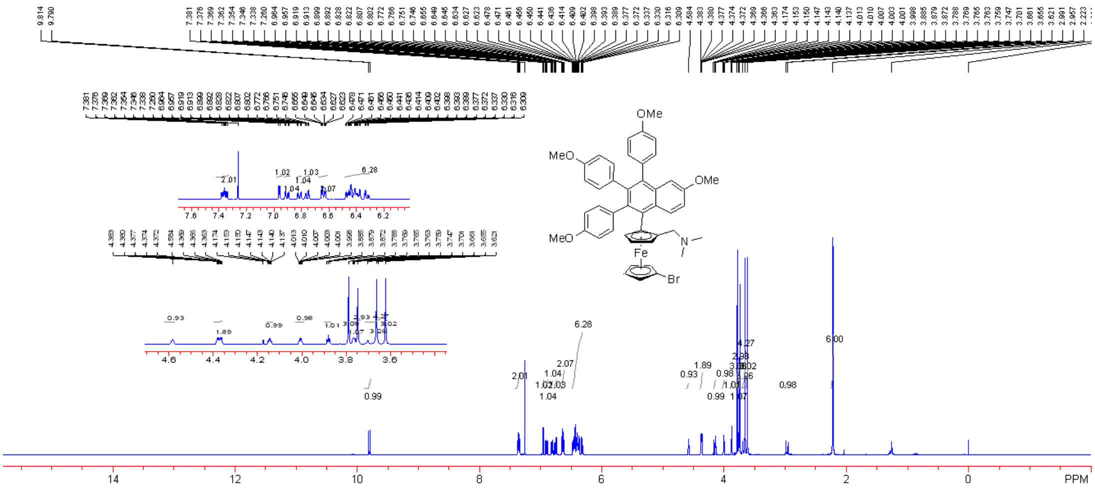
OMe
MeO
OMe
MeO
Fe
N
Br
6.28
6.00
PPM

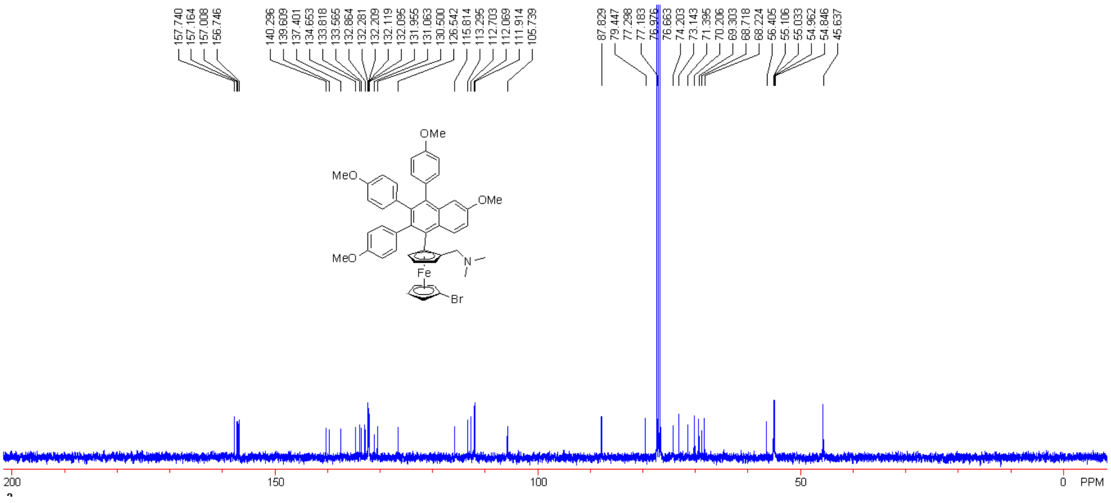
157.740
157.164
157.008
156.746
140.296
139.609
137.401
134.653
133.818
133.565
132.864
132.281
132.209
132.119
132.095
131.955
131.063
130.500
126.542
115.814
113.295
112.703
112.069
111.914
105.739
87.829
79.447
77.298
77.183
76.976
76.663
74.203
73.143
71.395
70.206
69.303
68.718
68.224
56.405
55.106
55.033
54.962
54.846
45.637
OMe
MeO
OMe
MeO
Fe
N
Br
PPM

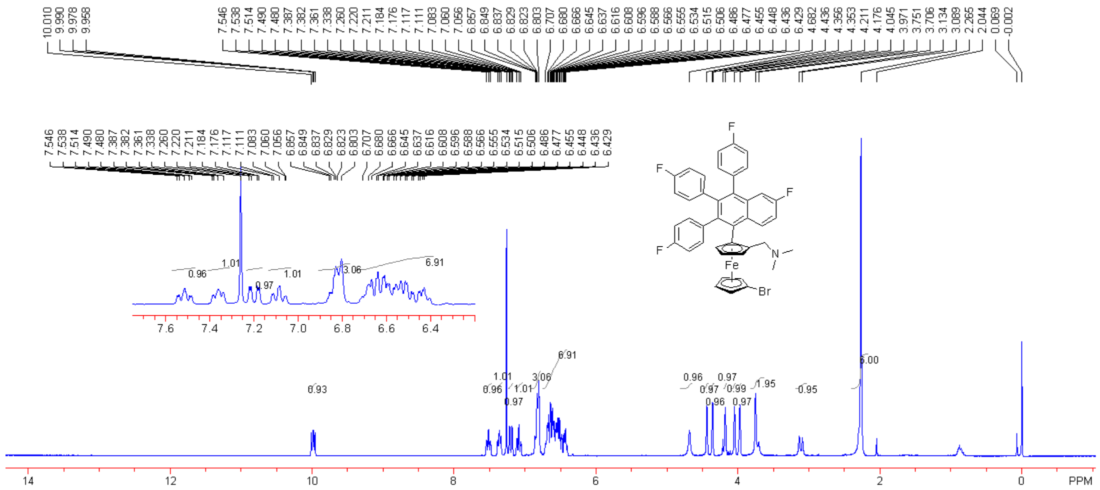

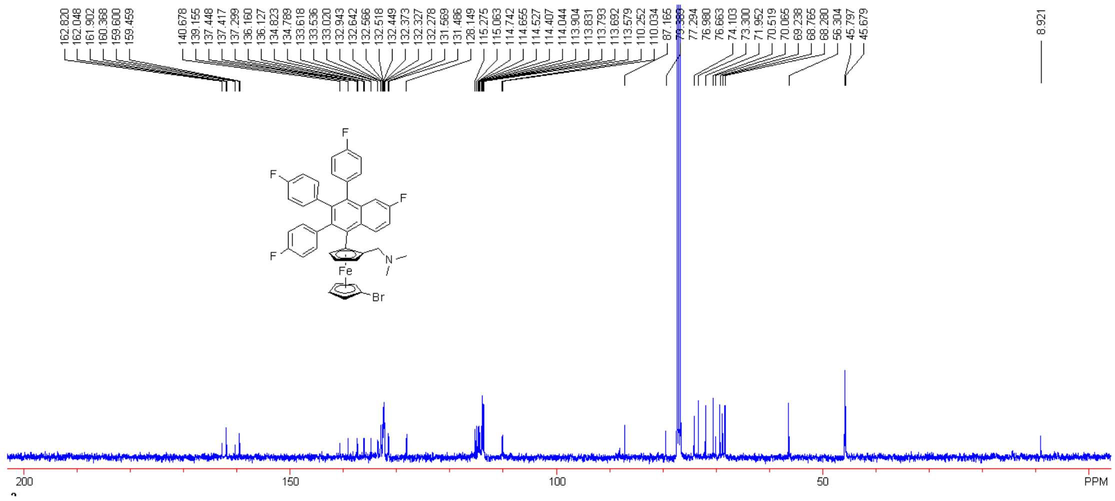

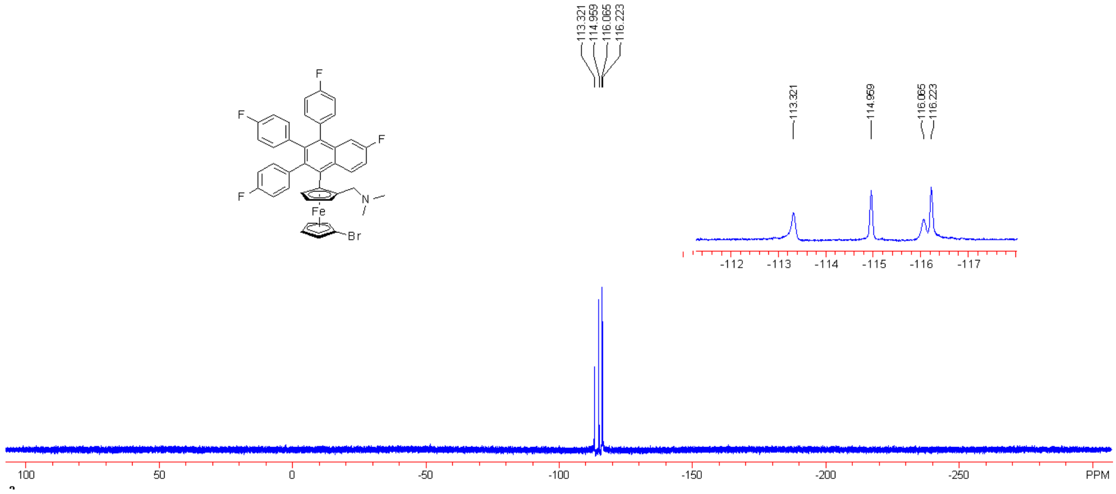

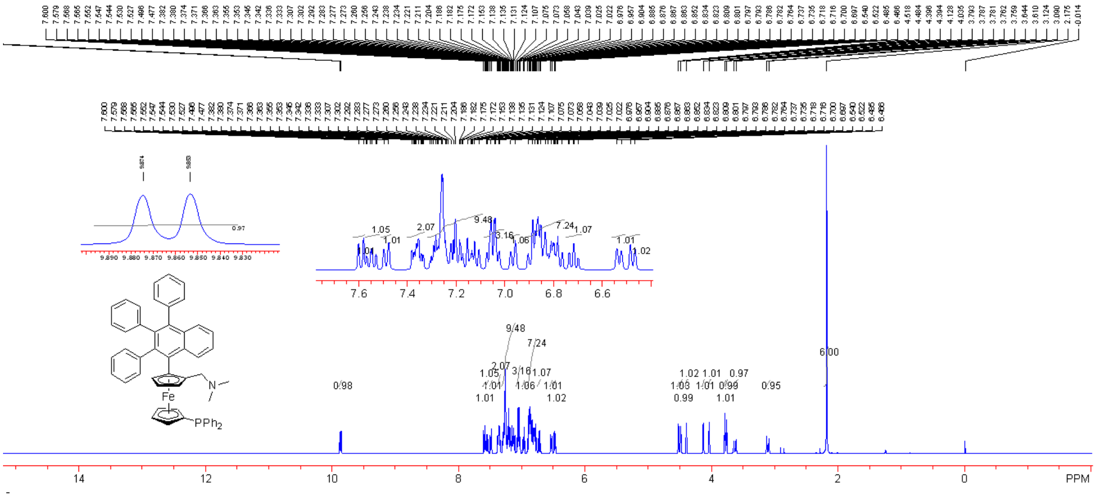

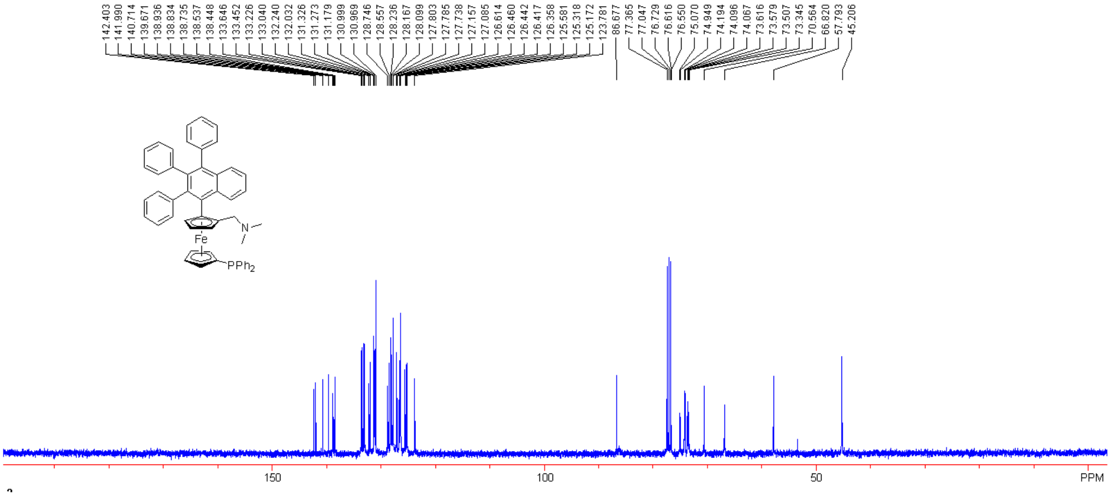

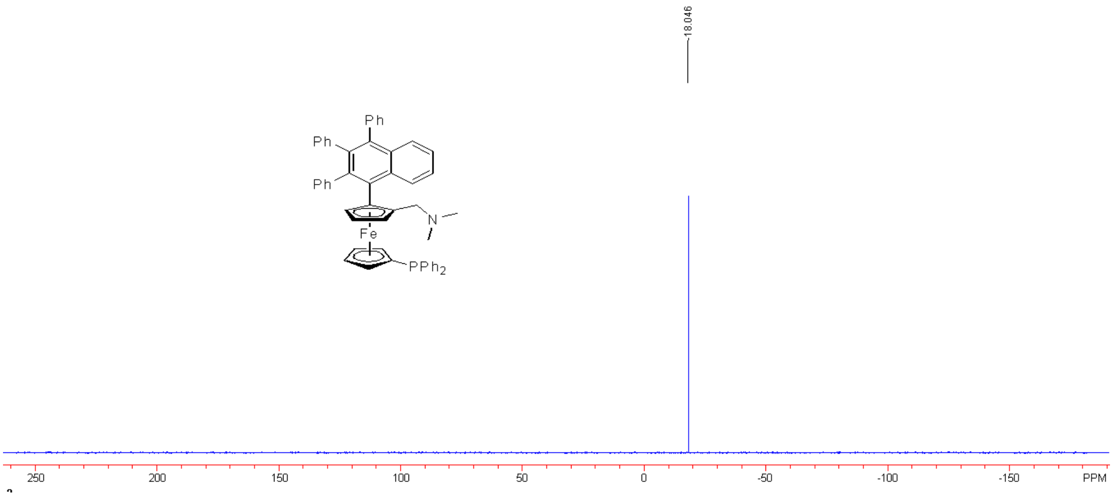
18.046
PPh2
PPM

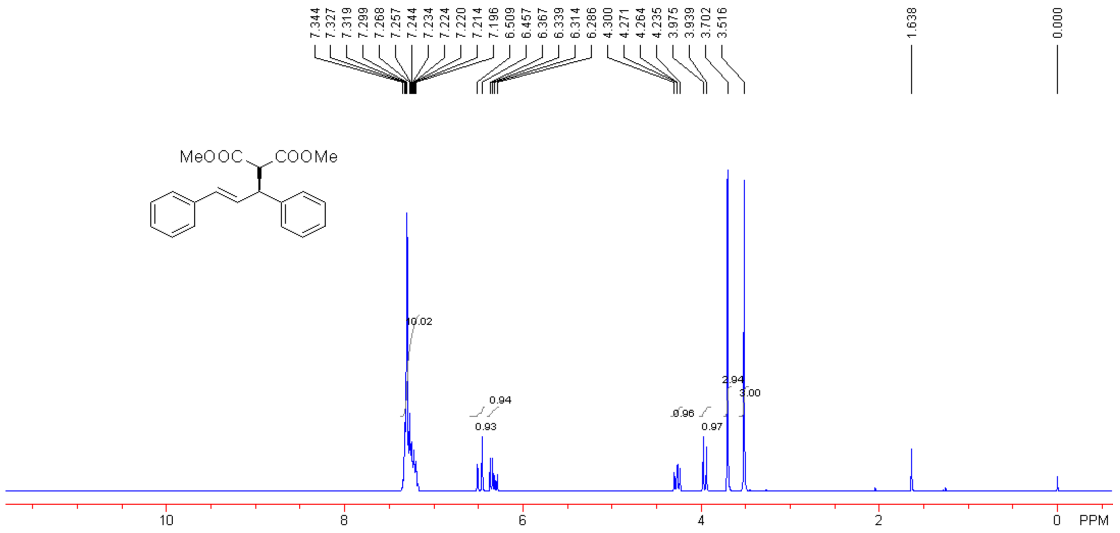

7.344 7.327 7.319 7.299 7.268 7.257 7.244 7.234 7.224 7.220 7.214 7.196 6.509 6.457 6.367 6.339 6.314 6.286 4.300 4.271 4.264 4.235 3.975 3.939 3.702 3.516
1.638
0.000
MeOOC
COOMe
10.02
0.94
0.93
0.96
0.97
2.94
3.00
10
8
6
4
2
0
PPM

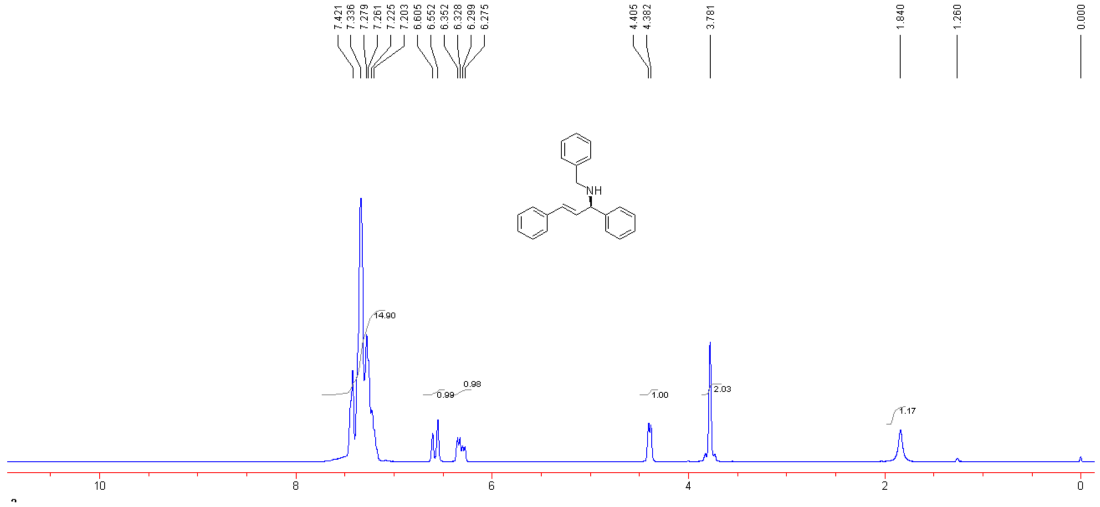

7.421 7.336 7.279 7.261 7.225 7.203 6.605 6.552 6.352 6.328 6.299 6.275
4.405 4.382
3.781
1.840
1.260
0.000
NH
14.90
0.98
0.99
1.00
2.03
1.17
10
8
6
4
2
0

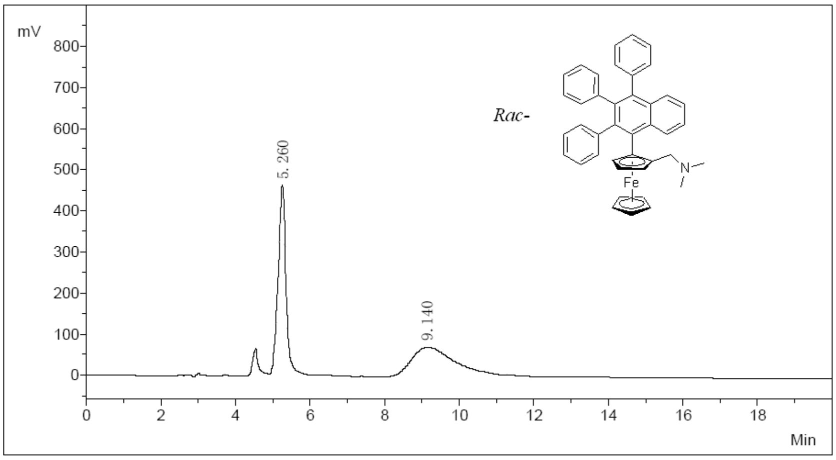

No.	PeakNo	ID.Name	R.Time	PeakHeight	PeakArea	PerCent
1	1		5.260	455177.3	6524683.9	51.0654
2	2		9.140	71564.6	6252421.1	48.9346
Total				526741.9	12777105.0	100.0000

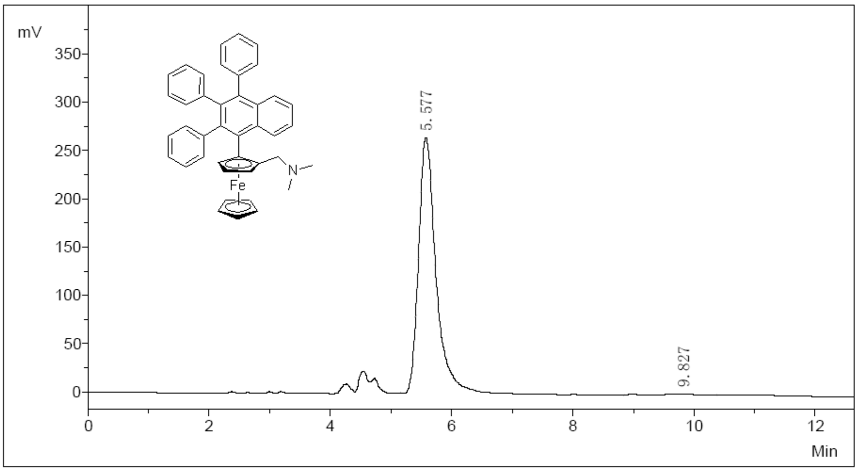

No.	PeakNo	ID.Name	R.Time	PeakHeight	PeakArea	PerCent
1	1		5.577	264324.1	5263154.8	99.0179
2	2		9.827	1280.4	52201.6	0.9821
Total				265604.5	5315356.5	100.0000

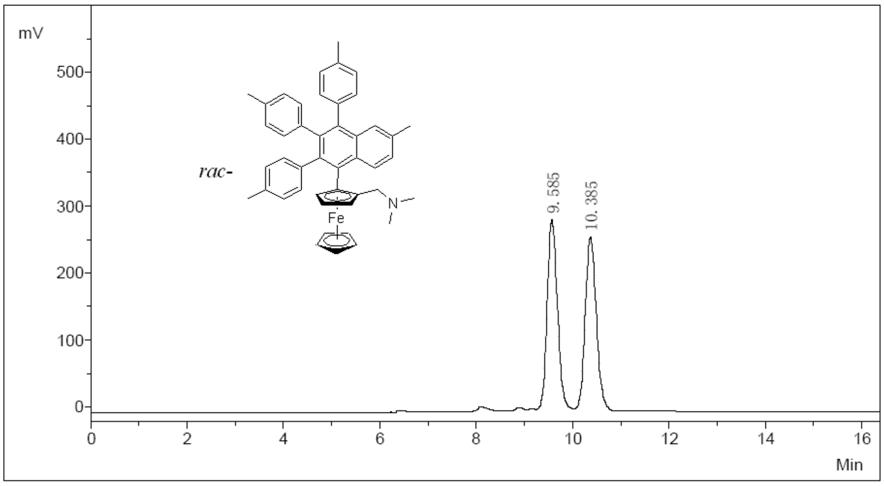

No.	PeakNo	ID.Name	R.Time	PeakHeight	PeakArea	PerCent
1	1		9.585	282080.5	4217669.7	50.9092
2	2		10.385	256623.8	4067027.2	49.0908
Total				538704.3	8284697.0	100.0000

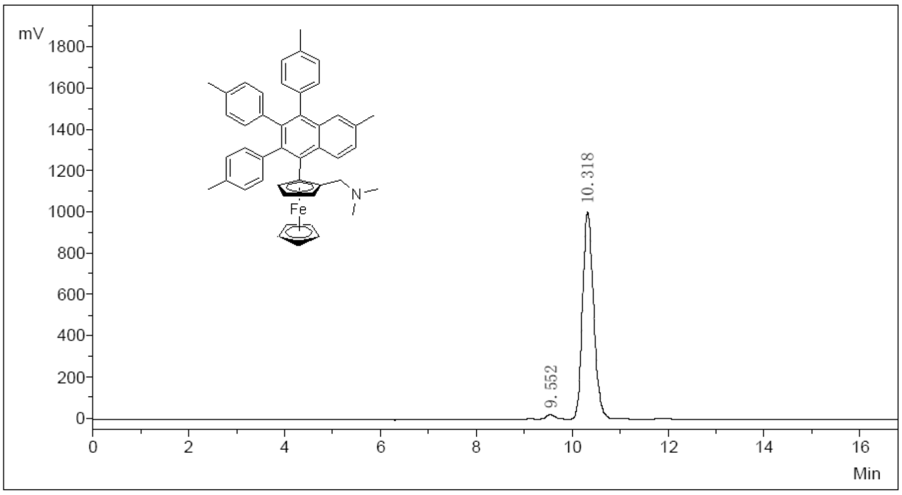

No.	PeakNo	ID.Name	R.Time	PeakHeight	PeakArea	PerCent
1	1		9.552	20173.1	283294.8	1.7260
2	2		10.318	1004494.0	16129919.7	98.2740
Total				1024667.0	16413214.5	100.0000

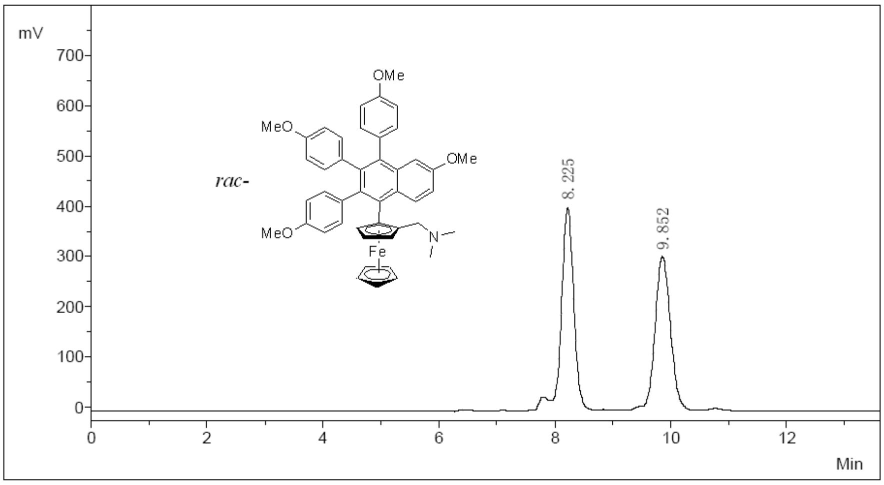

No.	PeakNo	ID.Name	R.Time	PeakHeight	PeakArea	PerCent
1	1		8.225	392907.1	5355204.4	48.5611
2	2		9.852	306171.9	5672562.1	51.4389
Total				699079.1	11027766.5	100.0000

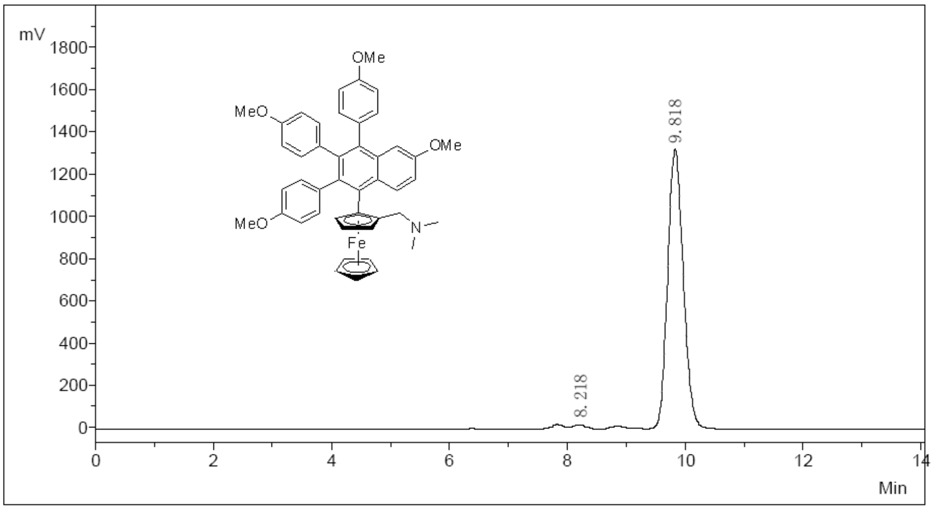

No.	PeakNo	ID.Name	R.Time	PeakHeight	PeakArea	PerCent
1	1		8.218	13115.0	143279.9	0.5833
2	2		9.818	1317328.3	24422418.8	99.4167
Total				1330443.3	24565698.7	100.0000

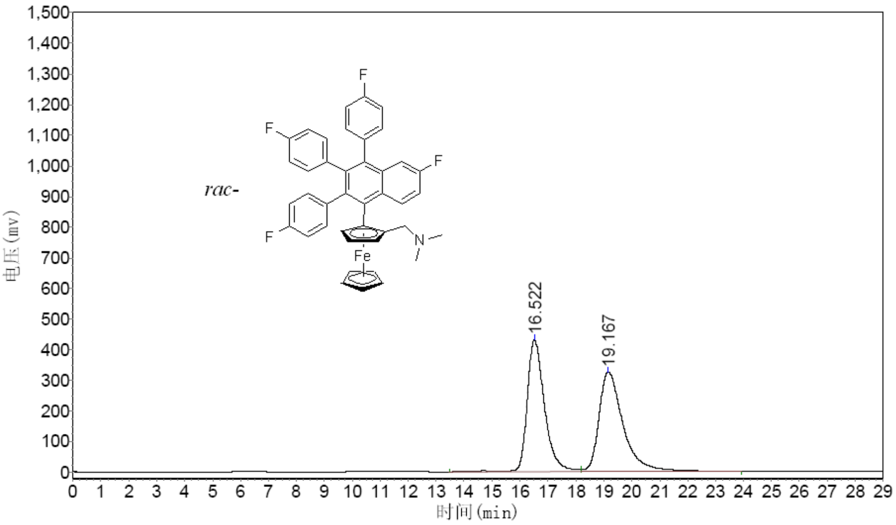

PeakNo	R.Time	PeakHeight	PeakArea	PerCent
1	16.522	432494.094	18804770.000	49.0590
2	19.167	325761.031	19526184.000	50.9410
Total		758255.125	38330954.000	100.0000

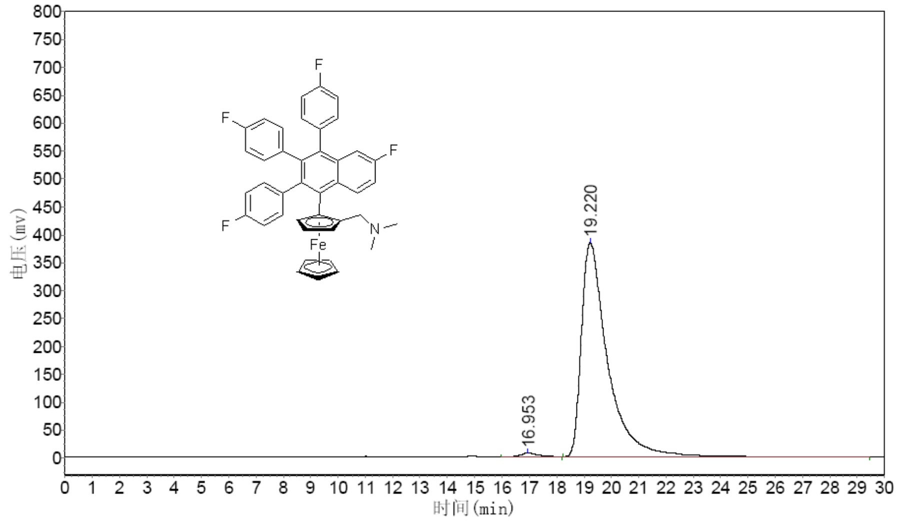

PeakNo	R.Time	PeakHeight	PeakArea	PerCent
1	16.953	6457.522	361496.344	1.3749
2	19.220	384619.594	25931188.000	98.6251
Total		391077.116	26292684.344	100.0000

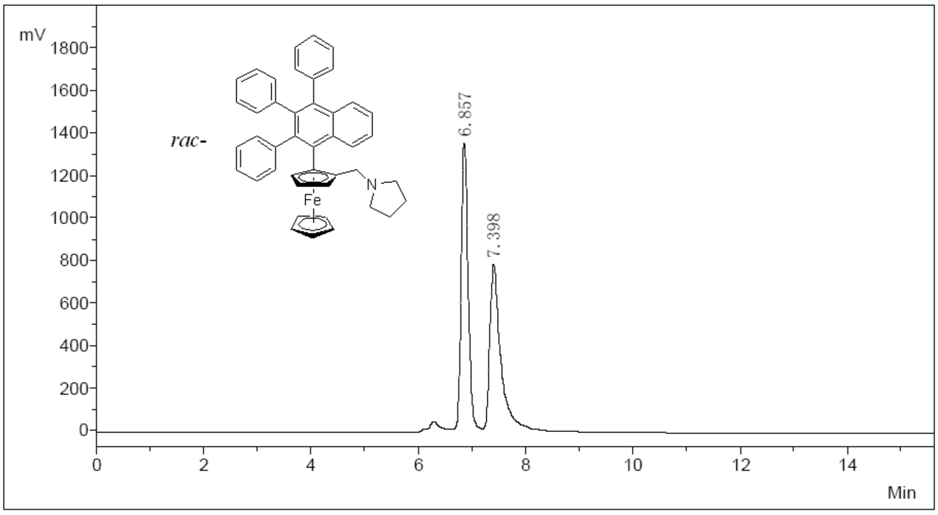

No.	PeakNo	ID. Name	R. Time	PeakHeight	PeakArea	PerCent
1	1		6. 857	1341416. 0	11818219. 4	51. 5591
2	2		7. 398	772948. 8	11103491. 1	48. 4409
Total				2114364. 8	22921710. 5	100. 0000

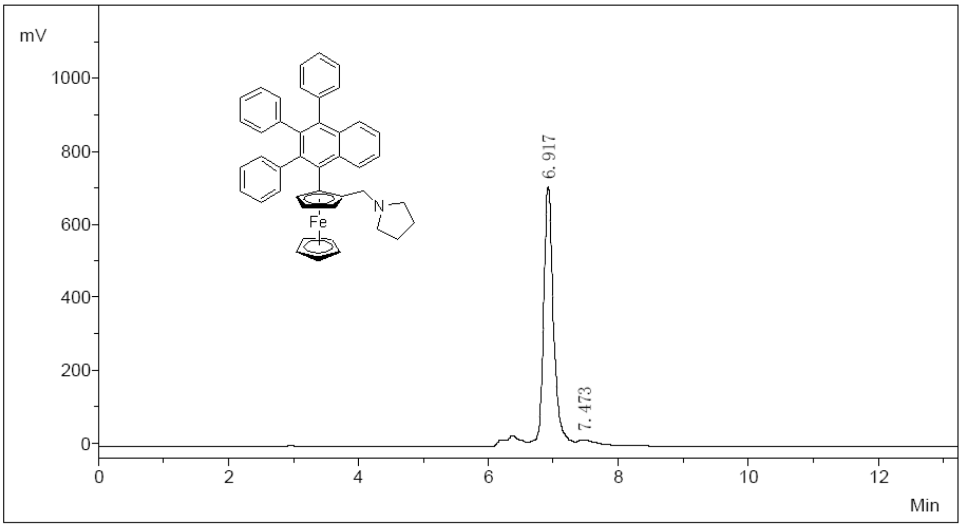

No.	PeakNo	ID. Name	R. Time	PeakHeight	PeakArea	PerCent
1	1		6.917	699828.0	7169651.8	97.6677
2	2		7.473	12231.9	171210.5	2.3323
Total				712059.9	7340862.4	100.0000

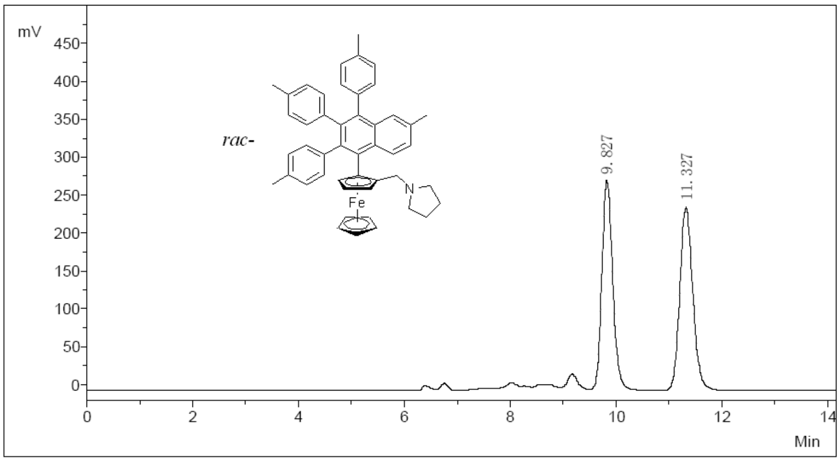

No.	PeakNo	ID.Name	R.Time	PeakHeight	PeakArea	PerCent
1	1		9.827	275220.9	3882593.2	48.7036
2	2		11.327	240766.1	4089283.3	51.2964
Total				515987.0	7971876.6	100.0000

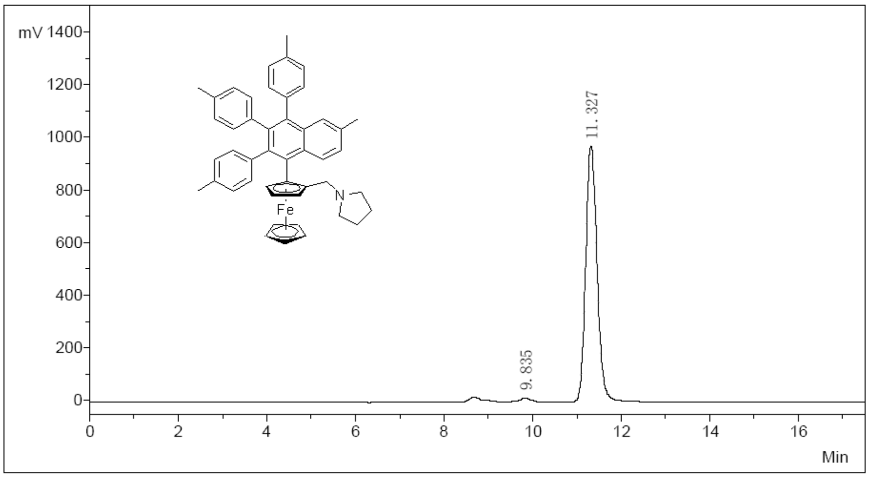

No.	PeakNo	ID.Name	R.Time	PeakHeight	PeakArea	PerCent
1	1		9.835	15306.8	247486.5	1.4074
2	2		11.327	970572.0	17337145.5	98.5926
Total				985878.8	17584632.0	100.0000

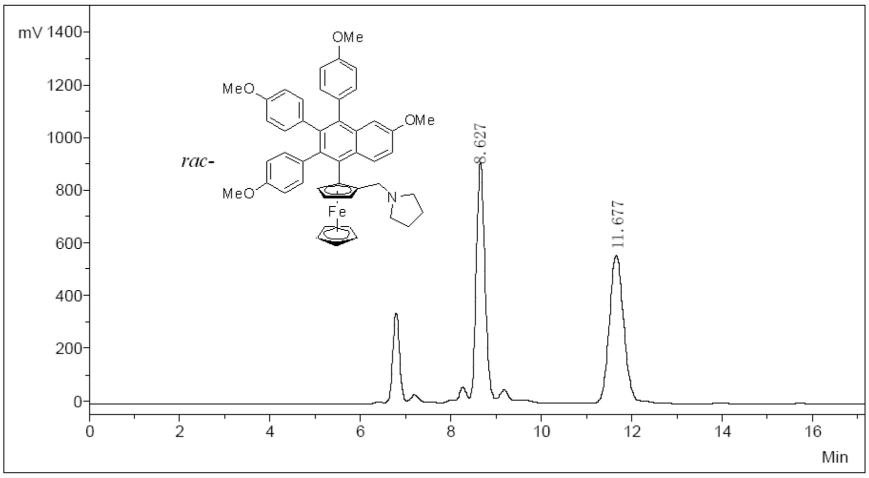

No.	PeakNo	ID. Name	R. Time	PeakHeight	PeakArea	PerCent
1	1		8.627	858645.1	11436145.9	48.8049
2	2		11.677	549721.2	11996248.2	51.1951
Total				1408366.3	23432394.1	100.0000

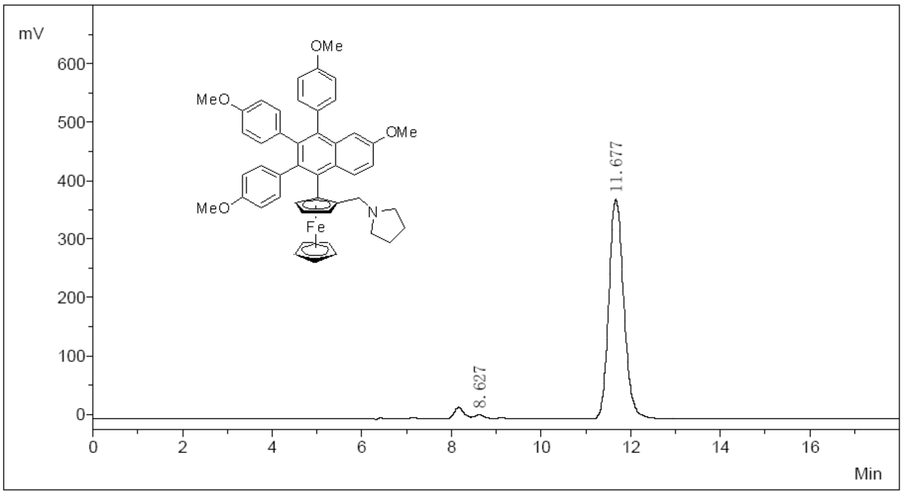

No.	PeakNo	ID. Name	R. Time	PeakHeight	PeakArea	PerCent
1	1		8.627	3837.3	45509.4	0.5188
2	2		11.677	375535.7	8725694.8	99.4812
Total				379373.0	8771204.2	100.0000

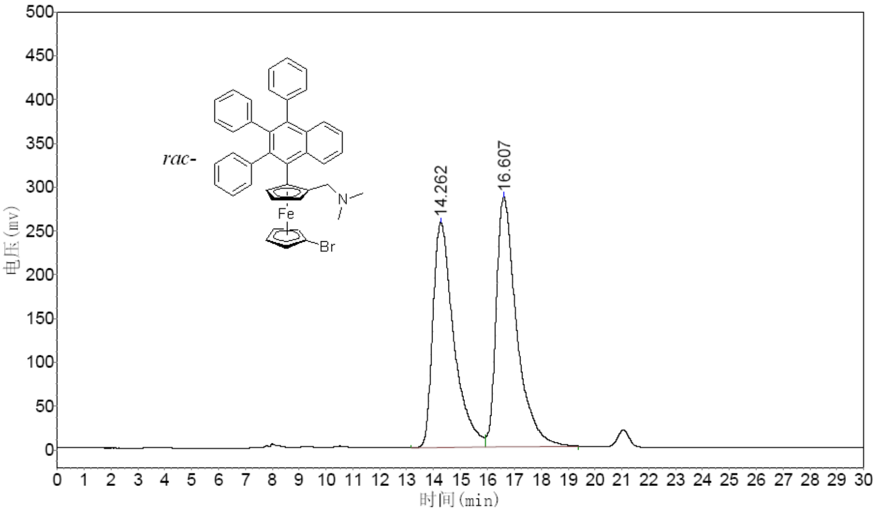

PeakNo	R.Time	PeakHeight	PeakArea	PerCent
1	14.262	257082.922	13896266.000	48.0055
2	16.607	285364.188	15050990.000	51.9945
Total		542447.109	28947256.000	100.0000

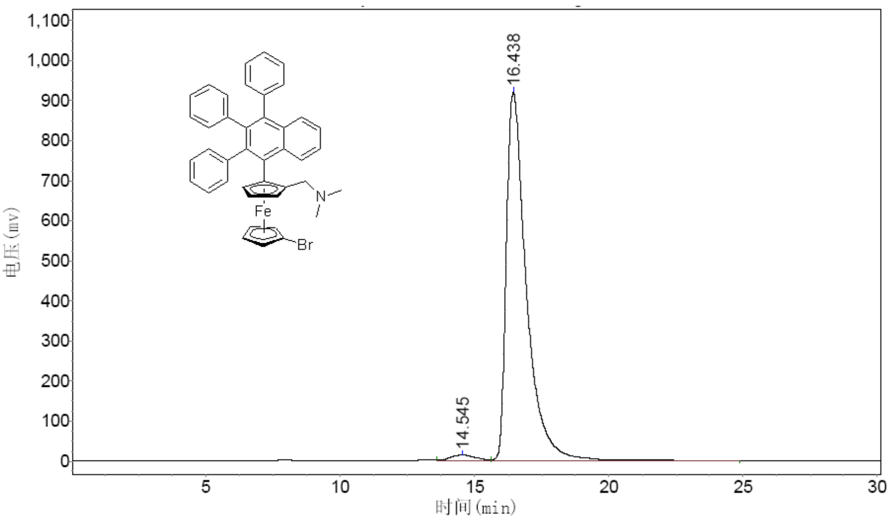

PeakNo	R.Time	PeakHeight	PeakArea	PerCent
1	14.262	257082.922	13896266.000	48.0055
2	16.607	285364.188	15050990.000	51.9945
Total		542447.109	28947256.000	100.0000

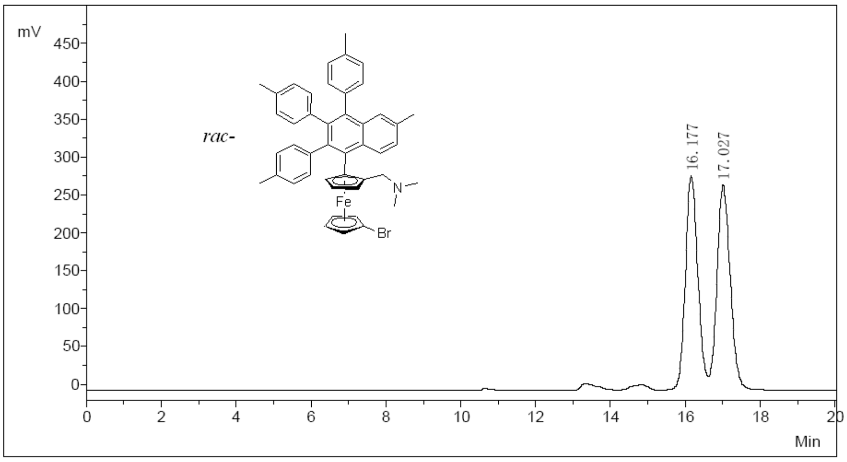

No.	PeakNo	ID.Name	R.Time	PeakHeight	PeakArea	PerCent
1	1		16.177	279914.6	6534434.8	49.2680
2	2		17.027	269295.8	6728597.1	50.7320
Total				549210.4	13263031.9	100.0000

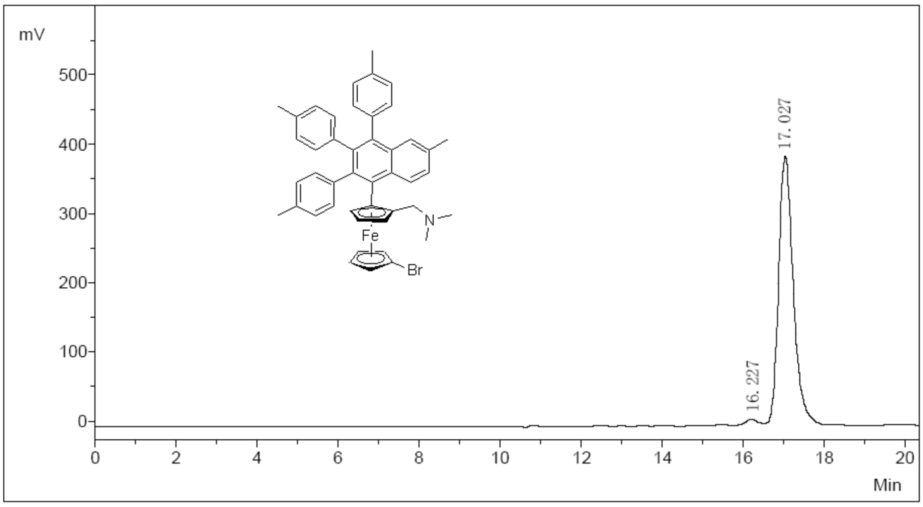

No.	PeakNo	ID.Name	R.Time	PeakHeight	PeakArea	PerCent
1	1		16.227	8941.5	213167.7	2.0942
2	2		17.027	388019.3	9965877.3	97.9058
Total				396960.7	10179045.0	100.0000

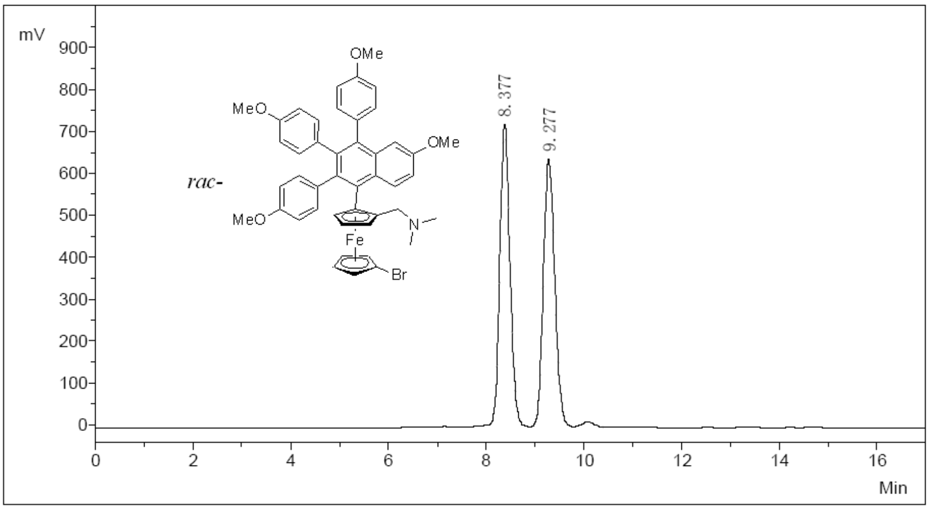

No.	PeakNo	ID.Name	R.Time	PeakHeight	PeakArea	PerCent
1	1		8.377	721269.0	10584821.6	50.4867
2	2		9.277	639616.7	10380726.0	49.5133
Total				1360885.7	20965547.6	100.0000

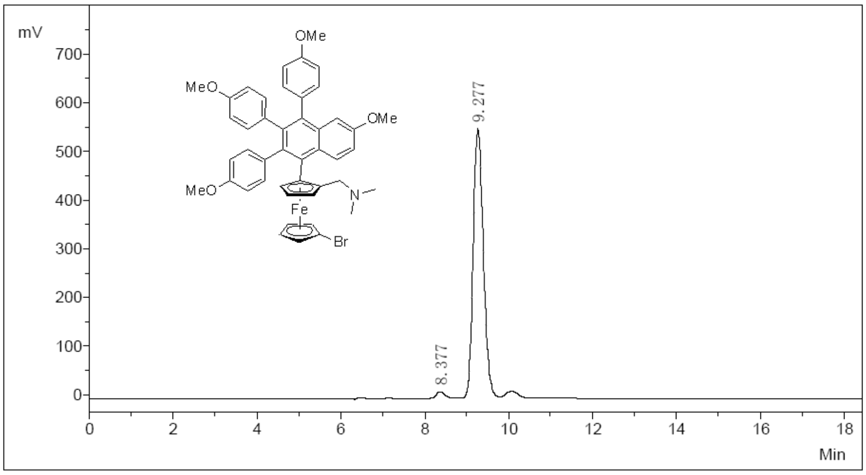

No.	PeakNo	ID.Name	R.Time	PeakHeight	PeakArea	PerCent
1	1		8.377	14377.1	208847.4	2.2047
2	2		9.277	550513.7	9264058.5	97.7953
Total				564890.8	9472905.9	100.0000

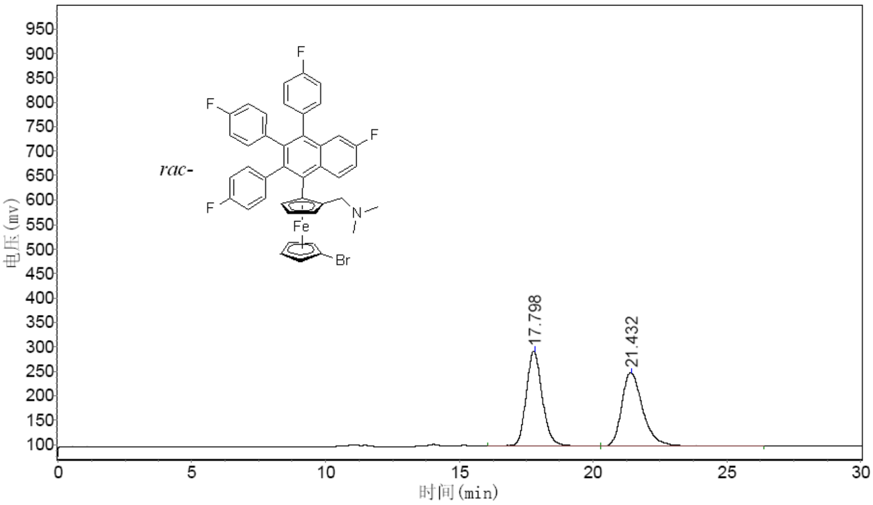

PeakNo	R. Time	PeakHeight	PeakArea	PerCent
1	17.798	195255.219	8292356.000	49.7119
2	21.432	150282.000	8388480.000	50.2881
Total		345537.219	16680836.000	100.0000

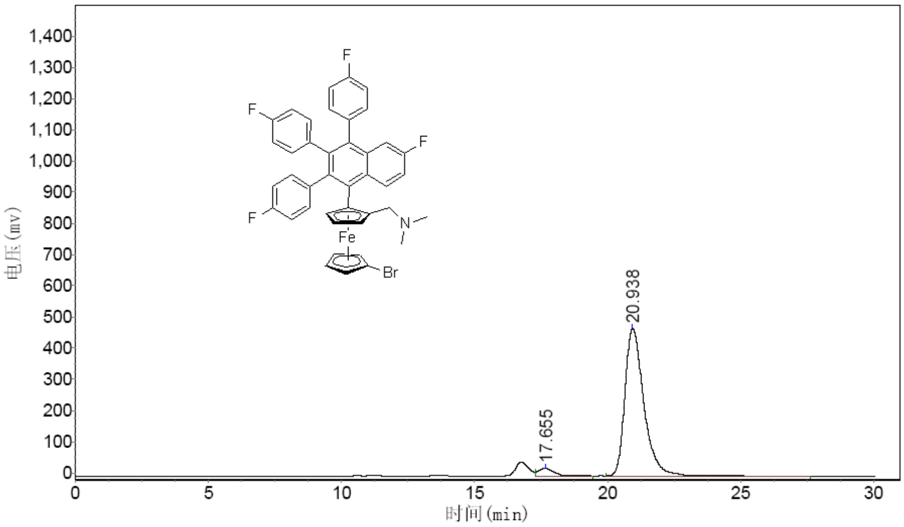

PeakNo	R. Time	PeakHeight	PeakArea	PerCent
1	17.655	25205.662	1033276.938	4.2002
2	20.938	471440.938	23567194.000	95.7998
Total		496646.600	24600470.938	100.0000

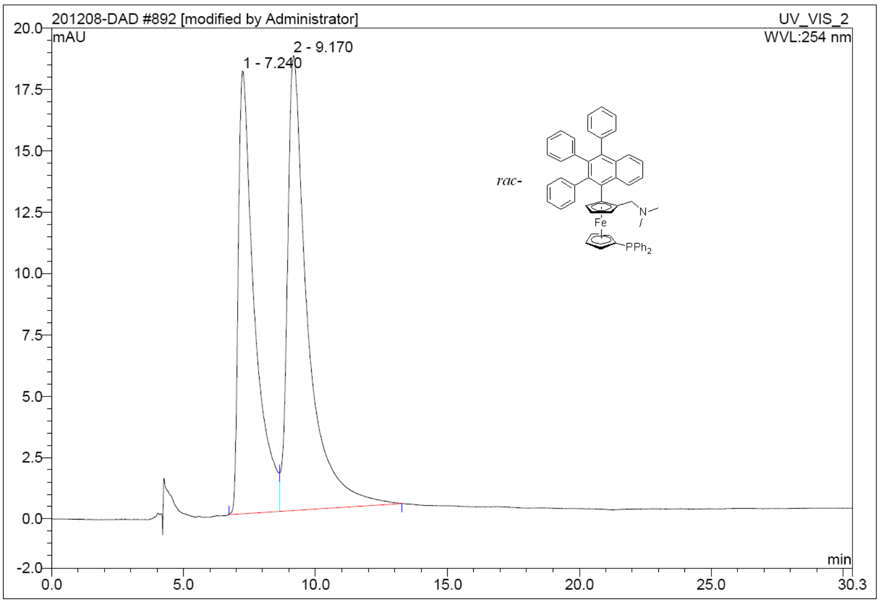

No.	Ret.Time min	Peak Name	Height mAU	Area mAU*min	Rel.Area %	Amount	Type
1	7.24	n.a.	18.048	12.783	43.62	n.a.	BM *
2	9.17	n.a.	18.536	16.521	56.38	n.a.	MB*
Total:			36.584	29.303	100.00	0.000	

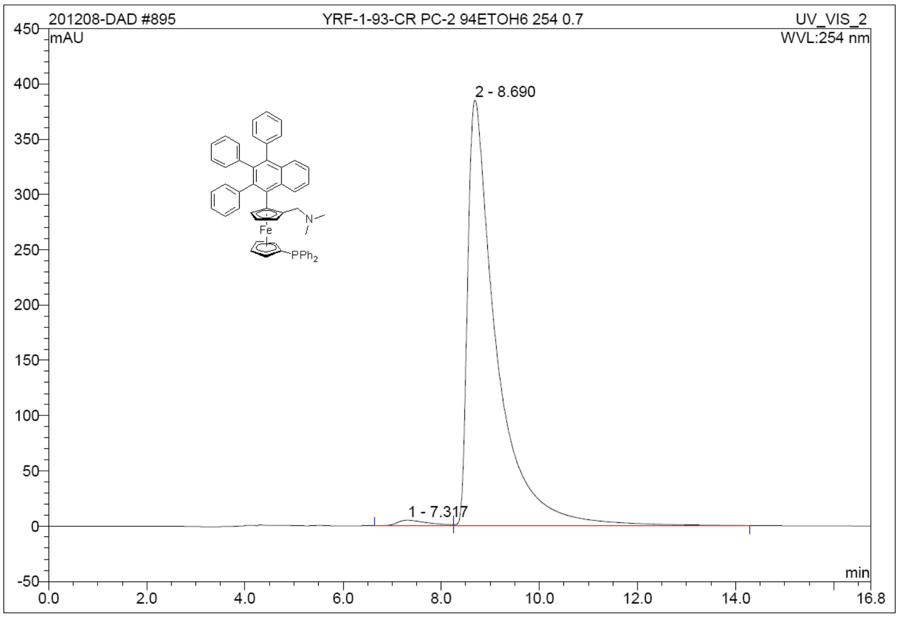

No.	Ret.Time min	Peak Name	Height mAU	Area mAU*min	Rel.Area %	Amount	Type
1	7.32	n.a.	4.900	3.551	1.35	n.a.	BM
2	8.69	n.a.	384.839	259.539	98.65	n.a.	MB
Total:			389.740	263.090	100.00	0.000	

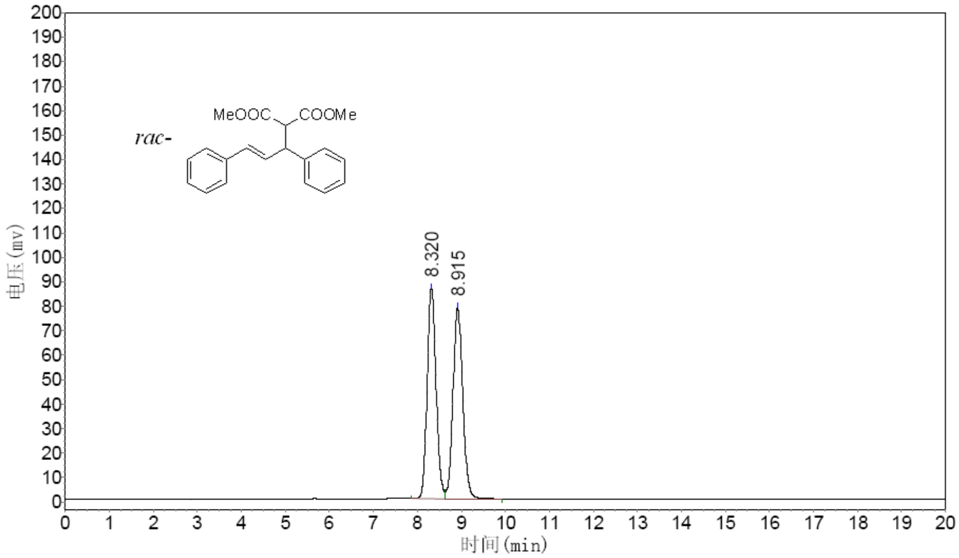

PeakNo	R. Time	PeakHeight	PeakArea	PerCent
1	8.320	85921.734	1200669.500	49.6353
2	8.915	78175.141	1218312.125	50.3647
Total		164096.875	2418981.625	100.0000

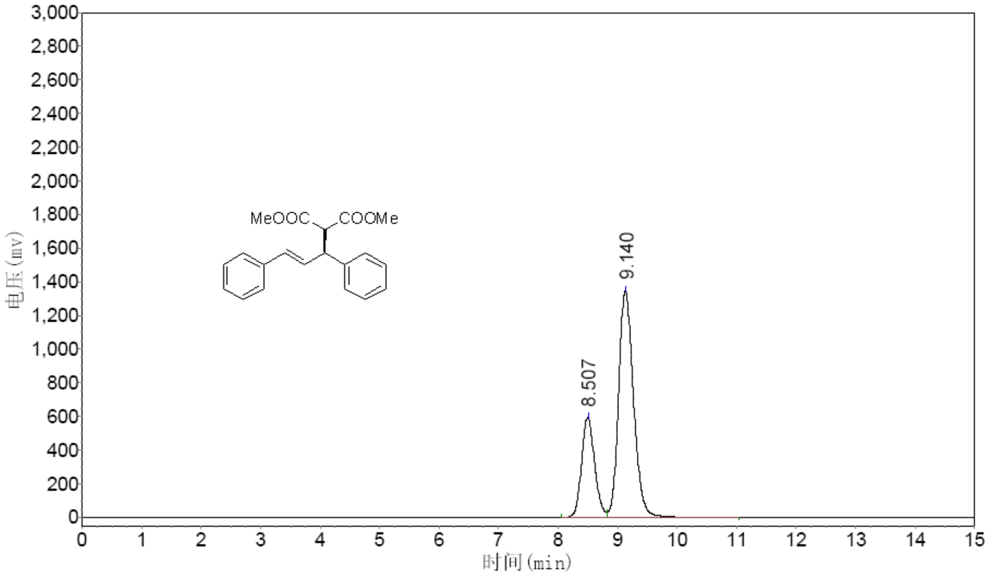

PeakNo	R. Time	PeakHeight	PeakArea	PerCent
1	8.507	591039.375	9057392.000	27.8780
2	9.140	1346444.000	23431976.000	72.1220
Total		1937483.375	32489368.000	100.0000

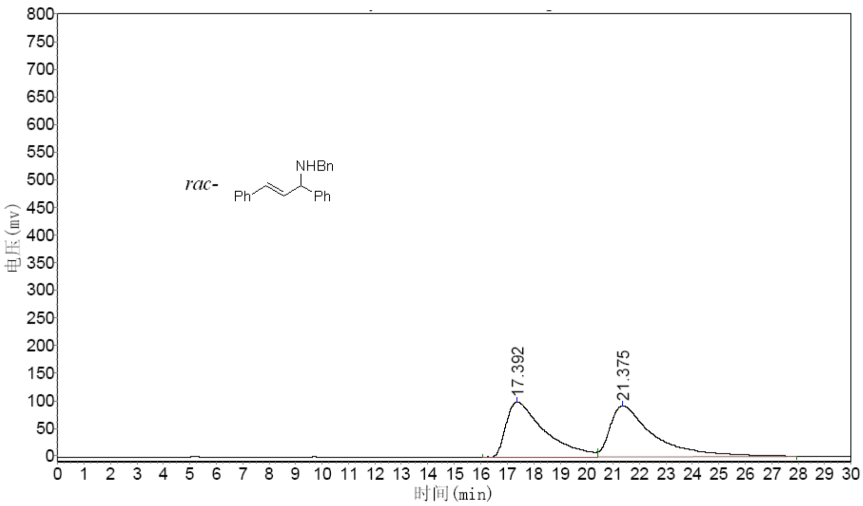

PeakNo	R. Time	PeakHeight	PeakArea	PerCent
1	17.392	99232.867	10057614.000	48.9027
2	21.375	92170.680	10508958.000	51.0973
Total		191403.547	20566572.000	100.0000

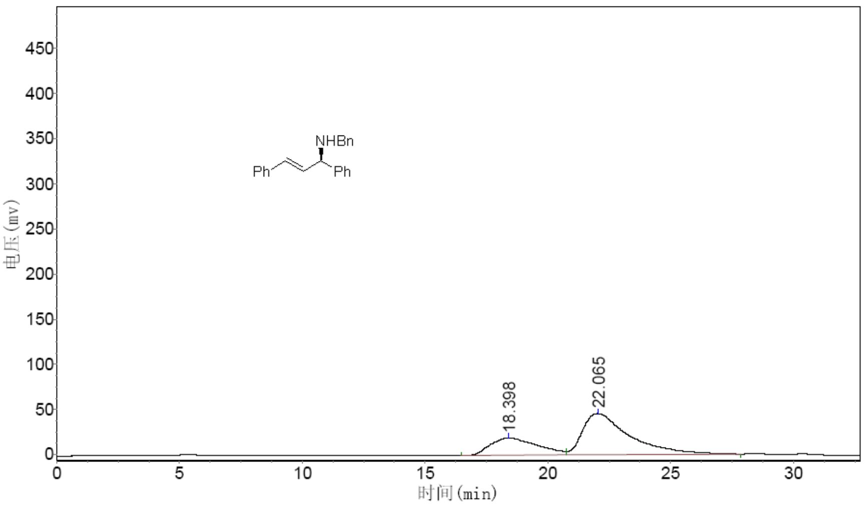

PeakNo	R. Time	PeakHeight	PeakArea	PerCent
1	18.398	19460.605	2660869.750	28.5714
2	22.065	46957.941	6652172.500	71.4286
Total		66418.547	9313042.250	100.0000
